# A kinome-wide screen identifies a CDKL5-SOX9 regulatory axis in epithelial cell death and kidney injury

**DOI:** 10.1038/s41467-020-15638-6

**Published:** 2020-04-21

**Authors:** Ji Young Kim, Yuntao Bai, Laura A. Jayne, Ralph D. Hector, Avinash K. Persaud, Su Sien Ong, Shreshtha Rojesh, Radhika Raj, Mei Ji He Ho Feng, Sangwoon Chung, Rachel E. Cianciolo, John W. Christman, Moray J. Campbell, David S. Gardner, Sharyn D. Baker, Alex Sparreboom, Rajgopal Govindarajan, Harpreet Singh, Taosheng Chen, Ming Poi, Katalin Susztak, Stuart R. Cobb, Navjot Singh Pabla

**Affiliations:** 1 Division of Pharmaceutics & Comprehensive Cancer Center, The Ohio State University, Columbus, OH USA; 2 Simons Initiative for the Developing Brain & Patrick Wild Centre, Centre for Discovery Brain Sciences, University of Edinburgh, Edinburgh, UK; 3 Division of Pharmacy Practice and Science, College of Pharmacy, The Ohio State University, Columbus, OH USA; 4 Department of Chemical Biology & Therapeutics, St. Jude Children’s Research Hospital, Memphis, TN USA; 5 Renal Electrolyte and Hypertension Division, Department of Medicine and Genetics, University of Pennsylvania, Philadelphia, PA USA; 6 Pulmonary, Sleep and Critical Care Medicine, Wexner Medical Center, Davis Heart and Lung Research Institute, Columbus, USA; 7 Department of Veterinary Biosciences, College of Veterinary Medicine, The Ohio State University, Columbus, OH USA; 8 School of Veterinary Medicine and Science, University of Nottingham, Loughborough, Leicestershire UK; 9 Department of Physiology and Cell Biology and Davis Heart and Lung Research Institute, The Ohio State University, Columbus, OH USA

**Keywords:** Stress signalling, Acute kidney injury

## Abstract

Renal tubular epithelial cells (RTECs) perform the essential function of maintaining the constancy of body fluid composition and volume. Toxic, inflammatory, or hypoxic-insults to RTECs can cause systemic fluid imbalance, electrolyte abnormalities and metabolic waste accumulation- manifesting as acute kidney injury (AKI), a common disorder associated with adverse long-term sequelae and high mortality. Here we report the results of a kinome-wide RNAi screen for cellular pathways involved in AKI-associated RTEC-dysfunction and cell death. Our screen and validation studies reveal an essential role of Cdkl5-kinase in RTEC cell death. In mouse models, genetic or pharmacological Cdkl5 inhibition mitigates nephrotoxic and ischemia-associated AKI. We propose that Cdkl5 is a stress-responsive kinase that promotes renal injury in part through phosphorylation-dependent suppression of pro-survival transcription regulator Sox9. These findings reveal a surprising non-neuronal function of Cdkl5, identify a pathogenic Cdkl5-Sox9 axis in epithelial cell-death, and support CDKL5 antagonism as a therapeutic approach for AKI.

## Abstract (web summary)

Protein kinases have emerged as critical regulators of disease pathogenesis. Here, the authors have utilized kinome-wide screening approaches to reveal a pathogenic role of CDKL5 kinase in acute kidney injury, which is dependent on suppression of a SOX9-associated transcriptional network.

## Introduction

The ability of vertebrates to maintain a stable, relatively constant “internal milieu” is inextricably linked to the function of the kidneys^1^. Through a continuous filtration–reabsorption process, kidneys regulate the fluid and molecular composition of blood. Within the kidneys, the renal tubular epithelial cells (RTECs) carry out the enormous task of selective reabsorption of water, ions, and essential nutrients, as well as excretion of metabolic waste, thereby converting the glomerular filtrate into a concentrated urine whose composition is constantly fine-tuned to maintain organismal homeostasis. RTEC dysfunction can thus lead to systemic electrolyte and fluid imbalances along with accumulation of metabolic and toxic waste triggering deleterious systemic effects and multi-organ failure.

Numerous clinical conditions such as sepsis, cardiac surgery, drug toxicities, cancer therapy, and rhabdomyolysis are associated with inflammatory, toxic, and hypoxic insults to RTECs^2–6^. The resulting RTEC dysfunction and cell death^7^ are the hallmarks and the underlying cause of acute kidney injury (AKI), a common disorder that predominantly develops in hospitalized patients^8^. Due to the lack of treatment options, annually an estimated 2 million people worldwide die of AKI^9^. Importantly, the patients that recover from an AKI episode are at increased risk of developing chronic kidney disease, end-stage renal disease, and cardiovascular dysfunction—disorders associated with significant morbidity and mortality^10,11^. Over the past decade, it has become apparent that the pathophysiology of AKI is exceedingly complex^12^. Multiple molecular and cellular pathways are involved in RTEC dysfunction and cell death^7^. Vascular and immune cells also contribute to renal impairment^13–15^. Recent advancements in our understanding of the pathophysiological basis of AKI have however not yet resulted in clinical benefits, in part, due to the non-druggable nature of several identified molecular targets and associated pathways. One possible way to transcend these difficulties is to utilize unbiased functional genomic screening to systematically uncover the role of “druggable genes” in AKI.

Of the estimated ~20,000 protein-coding genes in the human genome, ~10% encode proteins that can currently be targeted by small-molecule drugs, a group defined as “druggable genome”^16^. Protein kinases^17^ are one of the largest family in the “druggable genome”, along with G-coupled protein receptors. Due to the potential widespread role of kinases in disease pathogenesis, as well as suitable pharmacological properties and clinical safety profile of kinase inhibitors, protein kinases have emerged as attractive therapeutic targets^18,19^. Nevertheless, the underlying biology of the majority of kinases remains yet to be fully elucidated. Moreover, the role of protein kinases in the pathogenesis of non-oncological diseases, especially AKI, remains underexplored.

Here, we have used a kinome-wide screening approach to identify kinases that contribute to RTEC cell death in order to reveal therapeutic targets for AKI. Initial in vitro RNAi-based screening and subsequent in vivo validation experiments identified cyclin-dependent kinase-like 5 (Cdkl5) also known as serine/threonine kinase 9 (Stk9)^20^ as a key regulator of renal cell death and injury. *CDKL5* has mostly been studied for its role in human neuronal development since mutations in this *X*-linked gene are associated with neurodevelopmental disorders including early onset seizures^21,22^. Surprisingly, we have uncovered a previously unrecognized function of Cdkl5 as a crucial regulator of renal injury and identified the transcription factor Sox9 as one of its crucial downstream target.

## Results

### Identification of kinases involved in RTEC cell death

**Fig. 1:**
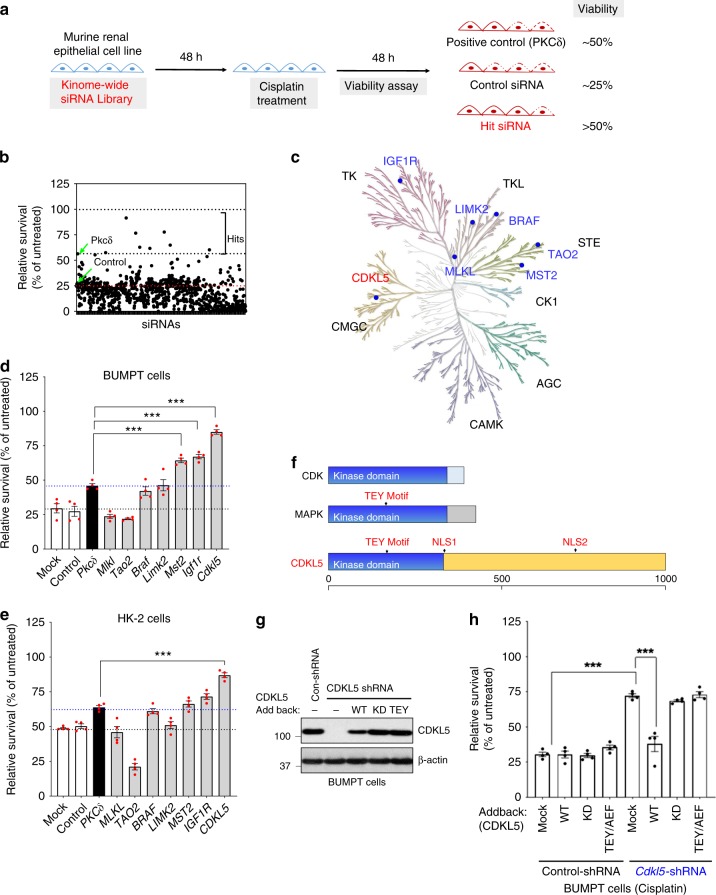
A kinome-wide screen uncovers protein kinases involved in RTEC cell death. **a** Scheme depicting the assay conditions used in the primary siRNA screen. BUMPT cells were transfected with Kinome-wide siRNA library (Dharmacon), followed by cisplatin treatment and cell-titer-glo-based viability assay. **b** Results of primary RNAi screening, shown by plotting the relative survival post cisplatin treatment of individual siRNA-targeted genes obtained from triplicate samples. **c** Kinome map (KinMap) depicting kinases identified in the primary screen. **d** Validation of primary hits by distinct siRNAs (Sigma) in BUMPT cells. Survival data (MTT assay) are presented as individual data points (*n* = 4 biologically independent samples), from one out of three independent experiments, all producing similar results. **e** Further secondary screening was carried out in HK-2 cells, by RNAi-mediated knockdown of indicates genes, followed by MTT-based cellular viability assay. Data are presented as individual data points (*n* = 4 biologically independent samples), from one out of three independent experiments, all producing similar results. **f** Schematic representation of CDKL5, the top hit and other members of CMGC kinase family. **g**, **h** Tertiary screening was carried for the top hit (*Cdkl5*) by shRNA-mediated knockdown in BUMPT cells and “add back” of wild-type and mutant *Cdkl5*. Cellular viability assays (MTT) showed that shRNA-mediated *Cdkl5* knockdown protects BUMPT cells from cisplatin-mediated cell death, an effect that was reversed by re-introduction of wild-type but not mutant *Cdkl5*. Data are presented as individual data points (*n* = 4 biologically independent samples), from one out of three independent experiments, all producing similar results. Representative western blot results demonstrating shRNA-mediated CDKL5 kinase knockdown and introduction of untagged wild-type, kinase-dead (KD), and TEY/AEF *Cdkl5* constructs. Data are representative of three independent experiments. In all the bar graphs, experimental values are presented as mean ± s.e.m. The height of error bar = 1 s.e. and *p* < 0.05 was indicated as statistically significant. One-way ANOVA followed by Dunnett’s (**d**, **e**) or Tukey’s multiple-comparison test (**h**) was carried out, and statistical significance is indicated by *p < 0.05, **p < 0.01, ***p < 0.001. Source data are provided as a Source Data file.

We performed a kinome-wide small-interfering RNA (siRNA) screen in BUMPT cells in order to identify protein kinases that regulate renal epithelial-cell death. High transient transfection efficiency (~95%) of this murine RTEC cell line makes it a suitable model for high-throughput (siRNA) screening assays. For the primary screen, BUMPT cells were transfected with either control siRNAs (non-targeting, *Pkcδ* and *Plk1*) or siRNAs targeting protein kinases, phosphatases, and related targets (780 genes, Dharmacon), followed by induction of cell death by treatment with cisplatin and assessment of cellular viability by cell-titer glo assay (Fig. 1a). Cisplatin-induced cell death in BUMPT cells partially mimics conditions observed during cisplatin-associated AKI^23^. The in vitro screening assay involved the treatment of BUMPT cells with 15 µM cisplatin, which reduced the cell viability by ~75% in 48 h in the untransfected and control siRNA- (non-targeting) transfected cells (Supplementary Fig. 1a, b). Cisplatin-induced cell death was partially ameliorated by protein kinase c *δ* (*Pkcδ*) knockdown (positive control), which is an established^24^ pro-apoptotic gene and significantly increased by polo-like kinase 1 (*Plk1*) knockdown (negative control).

The primary screen was carried out in triplicate, and subsequent data analysis (Fig. 1b, c) yielded seven hit candidates (Supplementary Table 1) that mitigated cell death to an extent that was significantly (*p* < 0.05, one-way ANOVA followed by Dunnett’s test) greater than the positive control (*Pkcδ* siRNA). For stringent validation of these identified hits, we performed confirmatory experiments by employing distinct siRNAs/shRNAs, cell lines, and assay systems. In the secondary screening, we utilized dissimilar siRNAs from a different source (Sigma) and used different cell viability and cell-death assays (MTT, Trypan Blue, and Caspase assay). Secondary screening in BUMPT cells (Fig. 1d and Supplementary Fig. 1c, d) validated three out of seven hits obtained in the primary screen. Similar studies in HK-2 (human kidney-2) cells, a human RTEC cell line showed that *CDKL5* knockdown significantly reduced cisplatin-induced cell death (Fig. 1e and Supplementary Fig. 1e, f). *Cdkl5* was the top hit in both the primary and secondary screens and hence we selected it for further confirmation.

The CDKL family (CDKL1–5) comprises five members that share structural similarities with CDKs as well as mitogen-activated protein kinases (MAPKs); however, their biological functions and linked signal transduction pathways remain obscure^25,26^. *CDKL5* is highly expressed in the brain and *CDKL5* loss-of-function mutations are associated with neurodevelopmental disorders in humans, although the underlying mechanisms are incompletely understood^27^. It also remains unknown if CDKL5 kinase controls any biological processes in nonneuronal tissues, such as testes and kidneys, where it is known to be expressed^20,28^.

Mechanisms underlying CDKL5 activation also remain unclear. However, similar to MAPKs, CDKL5 contains the TEY sequence within its activation loop (Fig. 1f). The TEY motif in the extracellular signal-regulated kinases (ERKs) undergoes dual phosphorylation resulting in kinase activation. This mechanism of activation is in most cases initiated by other upstream kinases or in some cases via autophosphorylation as has been proposed for ERK7 and CDKL5^29^. To confirm the role of Cdkl5 kinase in RTEC cell death, we carried out tertiary screening where we silenced *Cdkl5* expression in BUMPT cells using a shRNA targeting the 3′ UTR (untranslated region) of *Cdkl5* gene and carried out “add-back” experiments by overexpressing shRNA-resistant *Cdkl5* constructs, including wild-type, kinase-dead, and TEY mutants (Fig. 1g, h and Supplementary Fig. 1g, h). We found that shRNA-mediated *Cdkl5* knockdown reduces cisplatin-induced cell death, and importantly this phenotype was reversed by wild-type but not kinase-dead or TEY-mutant *Cdkl5* overexpression. Of note, overexpression of WT Cdkl5 in the control cells did not influence RTEC cell death. This may be due to limiting upstream activation signals, since unlike the wild-type Cdkl5, overexpression of catalytically active Cdkl5 (lacking the regulatory domain) increases cisplatin-associated RTEC cell death (Supplementary Fig. 1i–k). Collectively, our siRNA screening and validation studies identified Cdkl5 kinase (Fig. 1h) as a crucial, previously unknown regulator of renal epithelial-cell death.

### Cdkl5-kinase activity increases in RTECs during AKI

**Fig. 2:**
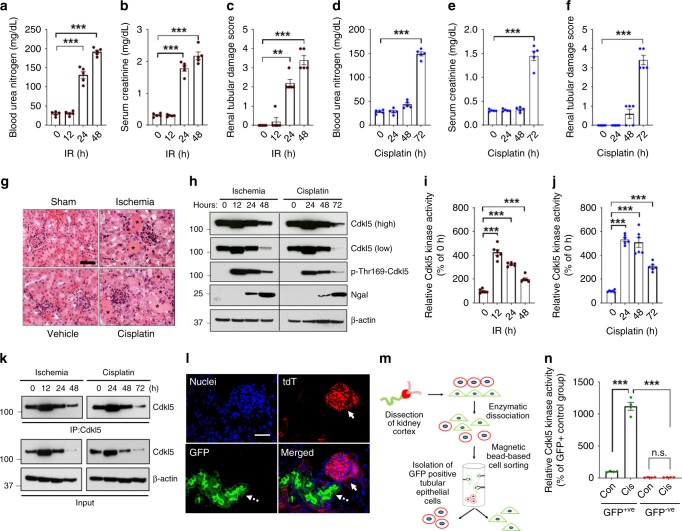
CDKL5 activity increases in renal tubular epithelial cells during AKI. **a**–**c** Bilateral renal ischemia was induced in male wild-type (C57BL/6) mice for 30 min followed by reperfusion for indicated timepoints. Blood urea nitrogen, serum creatinine, and histological analysis (H&E staining) were used to examine renal function and damage. **d**–**f** C57BL/6 mice were treated with cisplatin (30 mg/kg, intraperitoneal injection), and BUN, serum creatinine, and histological analysis were conducted at the indicated timepoints. **g** Representative H&E staining depicting tubular damage (indicated by asterisk) in both ischemic and cisplatin-treated mice. The graphs (**a**–**f**) represent data from a single experiment (*n* = 5 biologically independent samples), from one out of three independent experiments, all producing similar results. **h** Renal tissues from control, ischemic, and cisplatin-treated mice were used for western blot analysis of indicated proteins. Data presented are representative of five independent experiments, which yielded similar results. **i**–**k** Cdkl5 was immunoprecipitated from the kidneys of control, ischemic, and cisplatin-treated mice, followed by in vitro kinase assays. The representative western blots show the levels of Cdkl5 immunoprecipitated from tissue samples. The graphs represent data from a single experiment (*n* = 6 biologically independent samples), from one out of four independent experiments, all producing similar results. **l** *Ggt1-Cre* mice were crossed with *ROSA*^*mT/mG*^ mice to generate transgenic mice that express membrane-localized EGFP in renal tubular epithelial cells. A representative image shows EGFP expression in renal tubular cells. Arrows with dotted lines indicate tubular cells, while arrows with solid line show the glomerulus. **m** Schematic representation of the procedure used to isolate EGFP-positive renal epithelial cells. **n** Cdkl5 immunoprecipitation and in vitro kinase assay from indicated cells. The graph (*n* = 4) is representative of two independent experiments. In all the bar graphs, experimental values are presented as mean ± s.e.m. The height of error bar = 1 s.e. and *p* < 0.05 was indicated as statistically significant. One-way ANOVA followed by Dunnett’s (**a**–**f** and **i**, **j**) or Tukey’s multiple-comparison test (**n**) was carried out, and statistical significance is indicated by *p < 0.05, **p < 0.01, ***p < 0.001. Scale bar (**g** and **i**): 100 µm. Source data are provided as a Source Data file.

While we used a cisplatin-based in vitro screening method to identify putative regulators of RTEC cell death and dysfunction, our overall goal was to identify and validate targets that contribute to the pathogenesis of AKI associated with multiple etiologies. Hence, confirmatory in vivo studies were carried out in two distinct and widely used models of AKI, namely, ischemia–reperfusion injury and cisplatin-associated AKI^30^. In these mouse models, the onset of AKI was determined by three diverse indicators of renal structure and function: accumulation of nitrogenous waste (blood urea nitrogen and serum creatinine), biomarkers (kidney injury molecule-1 [*Kim1*]^31^ and neutrophil gelatinase-associated lipocalin [Ngal]^32^), and histological analysis (H&E staining and renal damage score) (Fig. 2a–g). In the ischemic injury model, AKI onset occurs 24-h post surgery, while in the cisplatin-associated renal injury model, renal impairment is seen 72-h post injection. We found that Cdkl5 protein levels showed significant variations, but overall, we observed marginal increase during the early phases of AKI, followed by reduction at later timepoints (Fig. 2h). To examine the Cdkl5 phosphorylation status in the activation loop, we generated a phospho-threonine-169 antibody that recognizes phosphorylated threonine within the TEY motif (Supplementary Fig. 2). Western blot analysis showed that Cdkl5 phosphorylation increased during AKI (Fig. 2h). Subsequently, kinase assays showed increased Cdkl5 activity in renal tissues during the early stages of AKI (Fig. 2i–k).

We next investigated whether the increased Cdkl5 activity is localized in the RTECs—the major cell type that is impacted during AKI^7^. In order to label and isolate RTECs from murine kidneys, we crossed the *ROSA*^*mT/mG*^ strain with the renal tubular epithelial-cell-specific *Ggt1-Cre* (gamma-glutamyltransferase-1) mice to generate transgenic mice that express membrane-localized green fluorescent protein (GFP) in the tubular epithelial cells (Fig. 2i and Supplementary Fig. 3). We then isolated GFP-positive cells from the kidneys of untreated and cisplatin-treated mice (Fig. 2m), followed by examination of Cdkl5 kinase activity (Fig. 2n). These studies confirmed that Cdkl5 activity increases in RTECs (GFP-positive cells) early during the development of AKI. Furthermore, increased Cdkl5-kinase activity was also observed in murine models of rhabdomyolysis and folic acid (FA)-associated AKI, as well as in a previously described^33^ porcine model of ischemic AKI (Supplementary Fig. 4a–g). In support of the in vivo studies, increased Cdkl5 activity was also observed in primary RTECs under multiple stress conditions, including cisplatin, hydrogen peroxide, hypoxia, and hemin treatments (Supplementary Fig. 4h, i). Under these conditions, increased Cdkl5 activity seemed to be independent of the cell-cycle phase. In summary, these results show that irrespective of the nature of the initial injury, an increase in Cdkl5 kinase activity is a common phenomenon during AKI, signifying a potential functional role in disease pathogenesis.

### Cdkl5 gene ablation in epithelial cells mitigates AKI

We next sought to examine the consequence of *Cdkl5* gene deletion on the severity of AKI. Germline *Cdkl5*-knockout mice are viable^27^, although they exhibit certain nonlethal neuronal phenotypes. We found that *Cdkl5*-knockout mice do not have any overt renal abnormalities under normal conditions (Supplementary Fig. 5a, b), which gave us the opportunity to examine the effect of *Cdkl5* deficiency on the severity of AKI. We found that as compared with wild-type littermates, *Cdkl5*^*−/y*^ mice showed protection from ischemia-associated AKI as revealed by multiple indicators: BUN, creatinine, *Kim1* expression, and histological analysis (Supplementary Fig. 6a–e). Likewise, *Cdkl5*^−^^*/y*^ mice displayed resistance to cisplatin-associated AKI (Supplementary Fig. 6f–i).

**Fig. 3:**
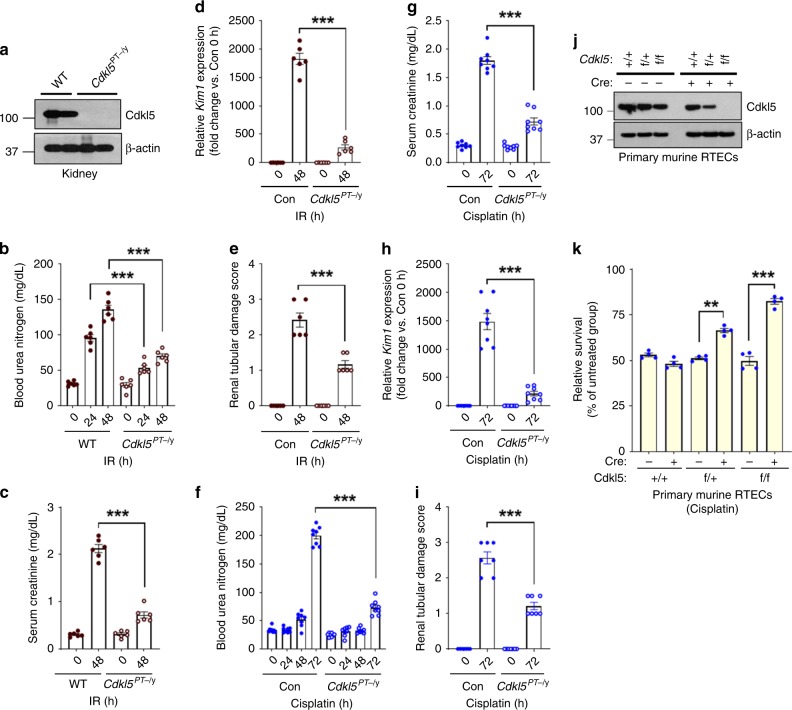
RTEC-specific Cdkl5 deletion provides protection from AKI. To generate mice with RTEC-specific *Cdkl5* knockout, *Ggt1-Cre* mice were crossed with *Cdkl5*-floxed mice. **a** Representative western blots showing successful knockout in the renal tissues. Littermate control and *Cdkl5* conditional knockout male mice (indicated by *Cdkl5*^PT−/y^) were then challenged with bilateral renal ischemia or cisplatin treatment. Bilateral renal ischemia was induced in wild-type and *Cdkl5*^PT−/y^ mice for 30 min followed by examination of renal structure and function. **b** Blood urea nitrogen, **c** serum creatinine, **d** renal *Kim1* mRNA expression, and **e** renal histological analysis (H&E) showed that tubular epithelial-specific *Cdkl5* deficiency confers protection from ischemia-associated AKI. Data presented (**b**–**e**) are cumulative of two independent experiments (*n* = 6). Wild-type and *Cdkl5*^PT−/−^ mice were treated with cisplatin (25 mg/kg) followed by examination of renal function. **f** Blood urea nitrogen, **g** serum creatinine, **h** renal *Kim1* mRNA expression, and **i** renal histological analysis (H&E) showed that *Cdkl5* contributes to cisplatin-mediated AKI. Data presented (**f**–**i**) are cumulative of two out of four independent experiments (*n* = 8) that showed similar results. **j** Primary renal tubular cells were cultured from female wild-type and *Cdkl5*-floxed mice. One week later, lentiviral transductions (Cre) were carried out to ablate *Cdkl5* gene. Western blot analysis confirmed CDKL5 ablation. Blots are representative of two independent experiments. **k** Primary renal tubular cells with indicated genotype were treated with 50 µM cisplatin, followed by cell viability assessment using trypan blue staining. Data are presented as individual data points (*n* = 4 biologically independent samples), from one out of three independent experiments, all producing similar results. In all the bar graphs, experimental values are presented as mean ± s.e.m. The height of error bar = 1 s.e. and *p* < 0.05 was indicated as statistically significant. One-way ANOVA followed by Tukey’s multiple-comparison test was carried out, and statistical significance is indicated by *p < 0.05, **p < 0.01, ***p < 0.001. Source data are provided as a Source Data file.

To probe the RTEC-specific role of Cdkl5 in the pathogenesis of AKI, we generated *Cdkl5* conditional knockout mice (*Cdkl5*^*PT−/y*^) by crossing the *Cdkl5*-floxed mice with the *Ggt1-Cre* mice. In *Ggt1-Cre* mice, *Cre* recombinase is expressed in RTECs 7–10 days after birth, such that *Cre* expression most likely occurs after the completion of renal development^34^. We found that normal renal function (Supplementary Fig. 5c, d) is unaffected by *Cdkl5* deficiency (Fig. 3a). Importantly, *Cdkl5* gene ablation in RTECs provided significant protection from ischemia-associated (Fig. 3b–e) and cisplatin-mediated (Fig. 3f–i) AKI. To investigate the effect of *Cdkl5* deficiency on renal cell death and to exclude the possibility of nonspecific compensatory changes, we cultured primary RTECs from the *Cdkl5*-floxed mice and carried out *Cdkl5* deletion under in vitro conditions using lentivirus-mediated *Cre* expression (Fig. 3j, k). We found that *Cdkl5* deletion provides significant protection from cisplatin-mediated cell death. Collectively, these studies suggested that Cdkl5 kinase plays a pathogenic role during the development of AKI.

### Cdkl5 phosphorylates Sox9 during AKI

**Fig. 4:**
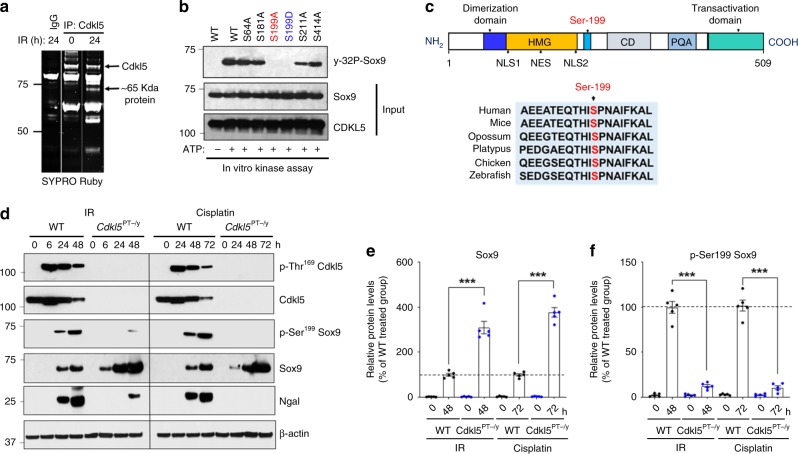
Cdkl5 phosphorylates Sox9 at Serine 199 site. **a** Bilateral renal ischemia was induced in C57BL/6 mice for 30 min followed by reperfusion for 1 day. Renal cortical lysates were then used to immunoprecipitate Cdkl5, while IgG was used as negative control. Immunoprecipitates were then run on a 4–12% gradient SDS-PAGE gel followed by protein visualization with SYPRO Ruby Protein Gel Stain. The ~65-kDa Cdkl5-interacting protein was then identified by mass spectrometric analysis as Sox9 as described in the “Methods” section. **b** Purified wild-type Cdkl5 and wild-type and mutant Sox9 proteins were co-incubated in a kinase assay buffer with [gamma-32P]-ATP for 30 min. Samples were then run on SDS-PAGE gel followed by transfer to PVDF membrane. Radiolabeled Sox9 was examined by autoradiography, followed by western blot analysis to examine the input proteins. Blots are representative of two independent experiments. **c** Schematic representation of Sox9 protein (modified from ref. ^64^). Protein sequence analysis showed that the sequence surrounding the Ser-199 site is highly conserved. HMG indicates high-mobility group box DNA-binding domain, CD indicates conserved domain, and PQA indicates proline–glutamine–alanine-rich domain. **d** Control, cisplatin, and ischemic renal tissues from control and *Cdkl5*^PT−/y^ mice were subjected to immunoblot analysis of indicated proteins. Blots are representative of at least three independent experiments. **e**, **f** Densitometric analysis of Sox9 and p-Ser-199–Sox9 protein levels. The graph represents cumulative results (*n* = 5 independent biological samples) from three independent experiments. Densitometric analysis was carried out using Image J, and the signals of indicated proteins were normalized by actin levels in the same samples. In all the bar graphs, experimental values are presented as mean ± s.e.m. The height of error bar = 1 s.e., and *p* < 0.05 was indicated as statistically significant. One-way ANOVA followed by Tukey’s multiple-comparison test was carried out, and statistical significance is indicated by *p < 0.05, **p < 0.01, ***p < 0.001. Source data are provided as a Source Data file.

We next pursued the Cdkl5-dependent mechanisms that contribute to renal dysfunction. CDKL5 regulates several neuronal functions; however, the downstream signaling pathways remain incompletely understood. Previous reports have described functional interactions of CDKL5 with other proteins with important neuronal functions^25,35–39^. Whether these interactions are relevant in renal epithelial cells is however unclear. Therefore, in an attempt to understand the mechanistic basis of Cdkl5-dependent renal injury, we sought to identify Cdkl5-interacting proteins. To this end, we immunoprecipitated (IP) endogenous Cdkl5 from ischemic renal tissues and found that an ~65-kDa protein was associated with Cdkl5. Mass spectrometric analysis identified this protein as the transcription factor Sox9 (sex-determining region Y (SRY) box 9) (Fig. 4a). Sox9 is a member of Sox family, which are a group of transcription factors that have essential roles in cell-fate determination during embryonic development and adult tissue homeostasis^40^. Interestingly, Sox9 is also known to suppress cell death during development, adult tissue homeostasis, and oncogenesis^41,42^.

We confirmed that Cdkl5 interacts with Sox9, by Cdkl5-IP and reverse IP (Sox9-IP) experiments (Supplementary Fig. 7). Notably, Sox9 protein is expressed at low amounts in control kidneys, and its expression is induced during AKI (input blots, Supplementary Fig. 7). Given the physical association between Cdkl5 and Sox9 in renal tissues, we considered if Sox9 is a previously unknown Cdkl5 substrate. Based on sequence analysis and global phospho-proteomics data^43^, we identified five putative phosphorylation sites in the Sox9 protein. We then tested the ability of purified Cdkl5 to phosphorylate wild-type and Ser-to-Ala Sox9 mutants. We found that Cdkl5 could phosphorylate wild-type Sox9 (Fig. 4b). Importantly, Ser-199 was found to be the major site of phosphorylation since Ser-to-Ala mutation at this site significantly abolished Cdkl5-mediated Sox9 phosphorylation (Fig. 4b). The Ser-199 site is evolutionarily conserved (Fig. 4c); however, the functional consequence of phosphorylation at this site has not been previously studied.

To ascertain the functional consequence of Cdkl5-mediated Sox9 phosphorylation, we investigated the potential effect of phosphorylation at Ser-199 site on Sox9 localization and stability. We generated S199A (non-phosphorylatable) and S199D (phospho-mimetic) Sox9 mutants and then examined their localization and stability in BUMPT cells. Sox9 subcellular localization was predominantly nuclear and was unaffected by S199A or S199D mutation (Supplementary Fig. 8a). Interestingly, cycloheximide (CHX) pulse-chase experiments showed that S199A mutant was more stable than the wild-type Sox9, while the phospho-mimetic S199D mutant had significantly reduced stability (Supplementary Fig. 8b, c). Based on these studies, we hypothesized that Cdkl5-dependent phosphorylation at Ser-199 suppresses Sox9 function during AKI.

To test our hypothesis and observe Sox9 phosphorylation in vivo, we generated an anti-phospho-Ser-199-specific antibody (Supplementary Fig. 9), and then examined the levels of total and phosphorylated Sox9 in renal tissues. In the wild-type mice, total Sox9 protein levels were low in control kidneys; however, its expression increased during both ischemia–reperfusion and cisplatin-associated AKI (Fig. 4d–f). Intriguingly, AKI-induced increase in the Sox9 protein expression had strikingly different dynamics in the *Cdkl5*^*PT−/y*^ mice. First, as compared with wild-type mice, AKI-associated Sox9 induction occurred at a much earlier timepoint in the *Cdkl5*^*PT−/y*^ mice, and second, the magnitude of Sox9 induction was higher in the *Cdkl5*^*PT−/y*^ mice. Interestingly, phospho-Ser-199–Sox9 levels also increased during AKI in the wild-type mice; however, Sox9 phosphorylation in the *Cdkl5*^*PT−/y*^ kidneys was pointedly suppressed (Fig. 4e, g). We also examined total and phosphorylated Cdkl5 protein levels in these tissues (Supplementary Fig. 10). Importantly, the levels of *Sox9* mRNA induction during AKI were not significantly different in the wild-type and *Cdkl5*^*PT−/y*^ mice (Supplementary Fig. 11). Based on these findings, we postulated that Cdkl5 activation might contribute to AKI, in part through phosphorylation-dependent regulation of Sox9 function.

### Sox9 plays a protective role during AKI

**Fig. 5:**
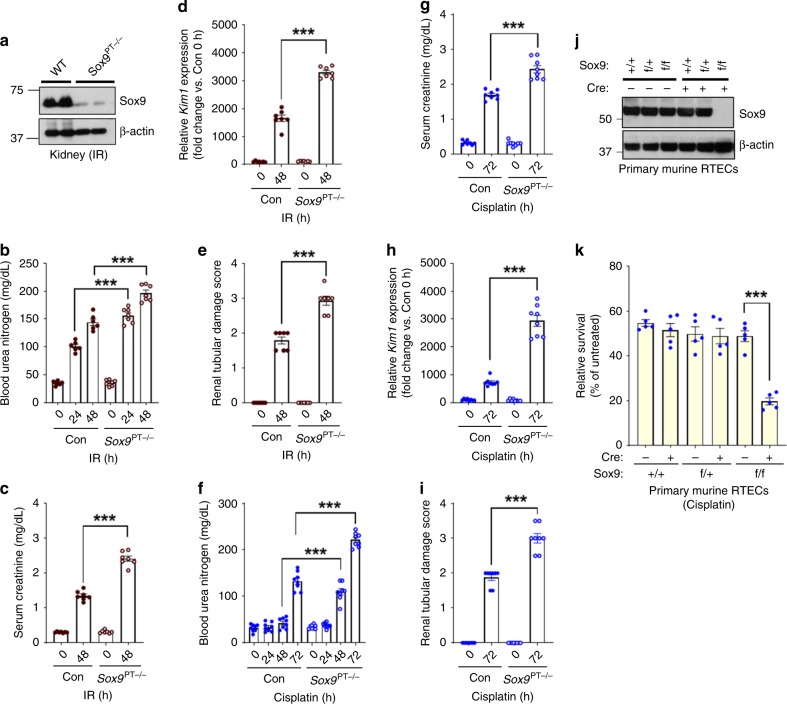
SOX9 plays a protective role during AKI. To generate mice with renal tubule-specific *Sox9* knockout, *Ggt1-Cre* mice were crossed with *Sox9-*floxed mice. **a** Representative western blots showing successful knockout in the renal tissues. Littermate control and *Sox9* conditional knockout mice (indicated by *Sox9*^PT−/−^) were used to study the role of SOX9 in AKI. Bilateral renal ischemia was induced in wild-type and *Sox9*^PT−/−^ mice for 30 min followed by examination of renal structure and function. **b** Blood urea nitrogen, **c** serum creatinine, **d** renal *Kim1* mRNA expression, and **e** renal histological analysis (H&E) showed that tubular epithelial-specific *Sox9* deficiency exacerbates ischemia-associated AKI. Data presented (**b**–**e**) are cumulative of three independent experiments (*n* = 6–7). Wild-type and *Sox9*^PT−/−^ mice were treated with cisplatin (30 mg/kg) followed by examination of renal function. **f** Blood urea nitrogen, **g** serum creatinine, **h** renal *Kim1* mRNA expression, **i** and renal histological analysis (H&E) showed that SOX9 regulates cisplatin-mediated AKI. Data presented (**f**–**i**) are cumulative of two out of four independent experiments (*n* = 8) that showed similar results. **j** Primary renal tubular cells were cultured from wild-type and *Sox9*-floxed mice. One week later, lentiviral transductions (Cre) were carried out to delete *Sox9* gene. Western blot analysis confirmed SOX9 deletion. Blots are representative of two independent experiments. **k** Primary renal tubular cells with indicated genotype were treated with 50 µM cisplatin, followed by cell viability assessment using trypan blue staining. Data are presented as individual data points (*n* = 4 biologically independent samples), from one out of three independent experiments, all producing similar results. In all the bar graphs, experimental values are presented as mean ± s.e.m. The height of error bar = 1 s.e. and *p* < 0.05 was indicated as statistically significant. One-way ANOVA followed by Tukey’s multiple-comparison test was carried out, and statistical significance is indicated by *p < 0.05, **p < 0.01, ***p < 0.001. Source data are provided as a Source Data file.

In the murine kidneys, Sox9 facilitates recovery of renal function after the onset of AKI^44,45^. After the initial injury phase, Sox9-expressing RTECs contribute to regeneration and recovery; however, the role of Sox9 in the initial injury phase remains unclear. To study the role of Sox9 in the early acute phase of AKI, we generated RTEC-specific *Sox9*-deficient (*Sox9*^*PT−/*^^−^) mice (Fig. 5a), which had normal renal function under baseline conditions (Supplementary Fig. 5e, f). Importantly, *Sox9* deficiency markedly increased renal damage in both the ischemia (Fig. 5b–e) and cisplatin-associated (Fig. 5f–i) AKI. Primary RTECs with *Sox9* gene ablation were also sensitive to cisplatin-mediated cell death (Fig. 5j, k). Interestingly, unlike the normal untreated kidneys (which have very low Sox9 expression), the primary RTECs expressed clearly detectable levels of Sox9 and were used for further studies. We carried out “add-back” experiments in the Sox9^−/−^ primary RTECs and found that S199A mutation provided significantly higher protection than the WT Sox9, while S199D mutant had minimal effects, which could be partly due to reduced S199D stability during cisplatin treatment (Supplementary Fig. 12). These results suggest that Sox9 plays a protective role during the early phase of AKI, and Cdkl5-mediated phosphorylation at S199 site likely reduces its functional activity.

To elucidate the underlying mechanisms, we next carried out chromatin immunoprecipitation (ChIP)-based analysis of Sox9 target genes in normal and injured kidneys (Supplementary Fig. 13a). Targets were selected based on ChIP-seq analysis in a previous study^46^ and included genes known to be differentially regulated during AKI^47^. Our results show that during ischemic injury, Sox9 binds to the promoter region (±2 kb of transcription start sites) of several genes (*Wwp2*, *Ap2β*, *Pi3kca, Myof, sema3e*, and *Gadd45a*). For *Wwp2, myof, Sema3e,* and *Gadd45a*, these findings were confirmed in three distinct models of AKI (Supplementary Fig. 13b–e). For further confirmation, gene expression analysis was carried out, which showed that as compared with the littermate controls, renal tissues of *Sox9*^*PT−/*−^ mice have diminished mRNA expression of *Wwp2*, *Myof*, *and Sema3e*, while *Gadd45a* expression is elevated (Supplementary Fig. 14). In the *Cdkl5*^*PT−/y*^ mice, which had elevated levels of Sox9 protein during AKI, the mRNA levels of Sox9-dependent pro-survival genes (*Wwp2, Myof, and Sema3e*) were significantly increased, while Gadd45a gene expression was reduced (Supplementary Fig. 15). Luciferase-based reporter assays confirmed Sox9 binding within the promoter regions of *Wwp2*, *Myof*, and *Sema3e* genes (Supplementary Fig. 16). Finally, functional studies show that *Wwp2, Myof, and Sema3e* knockdown sensitizes RTECs to injury, while *Gadd45a* knockdown provides protection from cell death (Supplementary Fig. 17). Thus, by increasing the expression of pro-survival genes like *Wwp2*, *myof*, and *sema3e*, Sox9 likely promotes cellular survival during AKI. These genes are known to regulate phosphoinositide 3-kinase (PI3K)—phosphatase and tensin homolog (PTEN) signaling (Wwp2)^48^, membrane and mitochondrial functions (Myoferlin)^49,50^, and cell death (Sema3e)^51^ in nonrenal epithelial cells. Whether these genes regulate RTEC dysfunction and cell death in vivo through similar mechanisms remains unknown. Notably, along with *Wwp2, Myof, and Sema3e*, Sox9-dependent renal protective transcriptional program likely involves multiple target genes that would require further exploration. However, our results support the notion that by suppressing Sox9 function, Cdkl5 subdues and delays a Sox9-dependent protective transcriptional program, contributing to epithelial-cell death and AKI.

### Targeted Cdkl5 inhibition mitigates renal injury in vivo

**Fig. 6:**
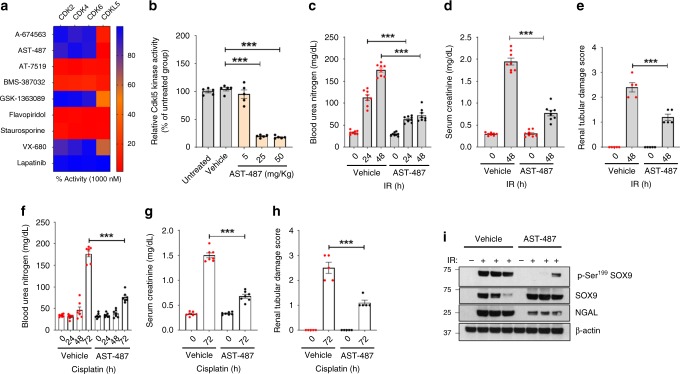
A small-molecule Cdkl5 inhibitor mitigates AKI. **a** In vitro kinase assays were carried out for cell-cycle-related kinases and CDKL5 for the indicated inhibitors at a single concentration of 1 µM. Kinase activity is presented as a heat map, where blue indicates no inhibition (high kinase activity), while red indicates kinase inhibition (low kinase activity). AST-487 was found to inhibit CDKL5, without affecting the activity of cell-cycle-related kinases. Data presented here are the mean of three independent experiments. **b** C57BL/6 mice were treated with either vehicle or AST-487 through oral administration followed by examination of Cdkl5 activity in renal tissues. Data are presented as individual data points (*n* = 5 biologically independent samples), from one out of two independent experiments, all producing similar results. **c**–**e** Bilateral renal ischemia was induced in wild-type C57BL/6 mice for 30 min followed by reperfusion for indicated timepoints. Mice were treated with either vehicle or AST-487 (25 mg/kg, oral gavage) 6 h post ischemia, followed by assessment of renal function and damage. **c** Blood urea nitrogen, **d** serum creatinine, and **e** renal histological analysis (H&E), Data presented (**c**–**e**) are cumulative of three independent experiments (*n* = 8). **f**–**h** Wild-type C57BL/6 mice were injected with cisplatin (30 mg/kg, i.p.) followed by treatment with either vehicle or AST-487 (25 mg/kg, oral gavage) 6 h later, followed by assessment of renal function and damage at indicated timepoints. Data presented (**e**–**h**) are cumulative of two out of four independent experiments (*n* = 8) that showed similar results. **i** Western blot analysis of renal tissues indicated that AST-487 suppresses Sox9 phosphorylation and increases Sox9 stability in vivo. Blots are representative of three independent experiments. In all the bar graphs, experimental values are presented as mean ± s.e.m. The height of error bar = 1 s.e. and *p* < 0.05 was indicated as statistically significant. One-way ANOVA followed by Dunnett’s (**b**) or Tukey’s multiple-comparison test (**c**–**h**) was carried out, and statistical significance is indicated by *p < 0.05, **p < 0.01, ***p < 0.001. Source data are provided as a Source Data file.

Genetic *Cdkl5* ablation alleviated renal injury, raising the prospect that a targeted Cdkl5-kinase inhibitor might prevent or reduce renal injury. While CDKL5-specific inhibitors have not been specifically pursued, several known protein kinase inhibitors have been tested for their ability to inhibit CDKL5 in global kinome-wide assays^52^. Based on these studies, we compiled a panel of small molecules with demonstrated CDKL5 inhibition activity. We then tested these compounds for their ability to inhibit Cdkl5 function using in vitro kinase assays (Fig. 6a). Among these inhibitors, AST-487 was found to be the most potent Cdkl5 inhibitor (EC_50_ = 87 nM). AST-487 also inhibited Cdkl5 activity in BUMPT cells and provided protection from cisplatin-induced cell death (Supplementary Fig. 18a–d). While AST-487 potently inhibited Cdkl5 activity, similar to most kinase inhibitors, AST-487 likely inhibits multiple kinases including RET kinase^53^. To examine the role of Cdkl5 inhibition in the renal protective effect of AST-487, we thus utilized a chemical genomics approach^54,55^. To this end, we generated a *Cdkl5* construct with a gatekeeper mutation (F89A), which confers resistance to AST-487-mediated kinase inhibition (Supplementary Fig. 18e). Importantly, overexpression of *Cdkl5*-gate-keeper mutant abrogated AST-487-mediated protection from cisplatin-induced cell death (Supplementary Fig. 18f–h). Since an AST-487-resistant Cdkl5 mutant is able to reverse the cytoprotective effects of AST-487, these studies provide compelling evidence that AST-487-mediated Cdkl5 inhibition is at least partly responsible for its renal protective effects.

To ascertain the potential efficacy of AST-487 in vivo, we performed pilot assessment studies. Oral administration of a single dose of 25 mg/kg AST-487 reduced Cdkl5-kinase activity in the kidneys by ~90% (Fig. 6b). Remarkably, AST-487 treatment (single dose of 25 mg/kg, 6 h after cisplatin injection or ischemic surgery) significantly reduced cisplatin and ischemia-associated AKI in the wild-type mice (Fig. 6c–h). We then carried out further studies in both control and *Cdkl5-*deficient mouse models. We found that AST-487 treatment reduced Cdkl5 phosphorylation and kinase activity (Supplementary Fig. 19a, b). Importantly, AST-487 treatment did not afford protective effects in the *Cdkl5*-deficient mice (Supplementary Fig. 19c–e). Furthermore, AST-487 treatment in wild-type mice resulted in blunted Sox9 phosphorylation and markedly increased accumulation of Sox9 during AKI (Fig. 6i and Supplementary Fig. 20). Even though AST-487 treatment conferred protection in the wild-type mice, we questioned if Cdkl5 inhibition just delays the development of kidney injury or it has long-term protective effects. Indeed, long-term survival experiments showed that AST-487 treatment reduces cisplatin-associated mortality (Supplementary Fig. 21a). In further support, genetic *Cdkl5-* deficiency also provides long-term protection and survival benefits (Supplementary Fig. 21b).

### Sox9-dependent and -independent regulation of AKI

**Fig. 7:**
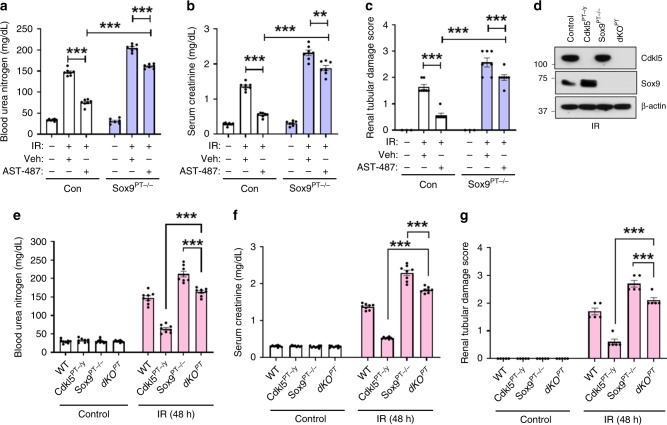
Cdkl5 regulates AKI in a Sox9-dependent and -independent manner. Bilateral renal ischemic surgery was carried out in littermate control and Sox9^PT−/−^ mice, followed by administration of either vehicle or AST-487 (25 mg/kg, oral gavage, 6 h post IR). At 48 h, renal function and damage were assessed through measurement of **a** blood urea nitrogen, **b** serum creatinine, and **c** renal histological analysis (H&E). Age-matched WT, Cdkl5^PT−/y^, Sox9^PT−/−^, and Cdkl5^PT−/y^Sox9^PT−/^^−^ (double-knockout mice indicated as dKO^PT^) underwent bilateral renal ischemia for 30 min, followed by **d** Western blot analysis of renal tissues at 24 h post reperfusion (one out of two independent experiments) and assessment of renal structure and function at 48 h through measurement of **e** blood urea nitrogen, **f** serum creatinine, and **g** renal histological analysis (H&E). Data presented (**a**–**c**, **e**–**g**) are cumulative of three independent experiments (*n* = 6). In all the bar graphs, experimental values are presented as mean ± s.e.m. The height of error bar = 1 s.e. and *p* < 0.05 was indicated as statistically significant. One-way ANOVA followed by Tukey’s multiple-comparison test was carried out, and statistical significance is indicated by *p < 0.05, **p < 0.01, ***p < 0.001. Source data are provided as a Source Data file.

To examine the dependence of Sox9 pathway in Cdkl5-associated renal injury, we initially examined the effect of Cdkl5 inhibition in control and *Sox9*-deficient mice challenged with ischemic injury. We found that Cdkl5 inhibition provides protection in both WT and *Sox9*^*PT−/−*^ mice; however, the extent of protection is much lower in the *Sox9*^*PT−/*−^ mice (Fig. 7a–c). Mice treated with cisplatin showed a similar phenotype (Supplementary Fig. 22a–c). We confirmed these results in primary RTECs, where Cdkl5 inhibition protected both WT and *Sox9*^−/−^ cells; however, the extent of protection was lower in the *Sox9*^−/−^ cells (Supplementary Fig. 23a–c).

To corroborate these findings, we next used the genetic knockout approach and performed similar studies in control, single-, and double-knockout mice (dKO^PT^) (Fig. 7d). As compared with the *Cdkl5*^*PT−/y*^ mice, the dKO^PT^ mice showed higher injury when challenged with ischemia, while as compared with the *Sox9*^*PT−/−*^ mice, the dKO^PT^ mice showed lower injury (Fig. 7d, g). We observed similar results in the cisplatin-toxicity model (Supplementary Fig. 22d–f). Studies with primary RTECs with single- or double-gene ablation also confirmed the in vivo findings (Supplementary Fig. 23d–g). These results suggest that Cdkl5 regulates renal injury in both Sox9-dependent and -independent manner. Furthermore, it is likely that regulation of Sox9 function during AKI occurs in both Cdkl5-dependent and -independent manner.

Finally, we performed a series of studies in female mice. We found that similar to male mice, Cdkl5 activity increases during AKI in females, and genetic or pharmacological inhibition of Cdkl5 function provides protection from ischemia and cisplatin-associated AKI (Supplementary Figs. 24 and 25). Cdkl5-dependent Sox9 phosphorylation was also confirmed in female mice (Supplementary Fig. 26). Collectively, these proof-of-principle experiments in multiple AKI mouse models showed robust therapeutic effects of Cdkl5 inhibition.

## Discussion

Here, we have found that Cdkl5 also known as serine/threonine kinase 9 (Stk9) is a stress–responsive kinase that controls epithelial-cell fate during AKI. We propose that Cdkl5 activation promotes renal dysfunction through phosphorylation-mediated functional suppression of pro-survival transcription factor Sox9.

Very little is known about the five members of the CDKL family (CDKL1–5), though they have been linked to certain neuronal functions^56^. In humans, mutations in the *X*-linked *CDKL5* gene are associated with neurodevelopmental disorders characterized by infantile seizures and developmental delay^22,35,57–60^. Some of these phenotypes have been recapitulated in the germline *Cdkl5*-knockout mice^27^. Most studies on CDKL5 function remain predominantly focused on its role in neuronal development. Interestingly, CDKL5 expression is not restricted to the brain, but is also detected in peripheral organs, particularly testes and kidney^20^. Our studies demonstrate Cdkl5 expression in RTECs and reveal its functional activation during AKI. It is noteworthy that germline or renal epithelial-cell-specific *Cdkl5* deficiency did not have any overt effect on normal kidney structure or function. Importantly, germline or RTEC-specific *Cdkl5* deletion conferred significant protection from AKI. Primary RTECs with *Cdkl5* deficiency were also resistant to cellular injury. These studies suggest that Cdkl5 is not required for normal renal development or function; however, under stress conditions, Cdkl5 contributes to renal cell death and dysfunction.

The CDKL family shares structural features with CDKs as well as MAPKs and glycogen synthase kinases^56^. Although their nomenclature suggests similarity with CDKs, CDKLs have several features that distinguish them from CDKs, including the lack of evidence that CDKLs require cyclin binding, the presence of variant PSTAIRE motifs within the C helix, and large C-terminal regulatory domains with nuclear localization signals^56^. Moreover, there is no clear evidence that CDKLs are involved in cell-cycle regulation. Interestingly, our studies suggest that Cdkl5 might be a cell-cycle-independent stress–responsive kinase in RTECs, with much more functional similarity with MAPKs than CDKs. In support of this notion, our studies show Cdkl5 activation under markedly distinct conditions of cellular stress both in vitro and in vivo. In this regard, Cdkl5 seems to share functional similarities with MAPKs, which are known components of cellular stress response pathways^61^.

While the upstream signaling remains unknown, we have identified the transcription factor Sox9 as a bona fide Cdkl5 substrate and a key downstream target in renal epithelial cells. The endogenous substrates of CDKL5 have been previously investigated to understand its function in neurons^25,35–39^. Whether these previously identified substrates are involved in Cdkl5-dependent renal cell death remains unclear. However, through a pull-down experiment, we identified Sox9 as a Cdkl5 substrate in RTECs. Sox9 is a transcription factor that controls cell-fate decisions during embryonic development and homeostasis of a broad range of adult tissues^62–64^. Moreover, in cancer cells, SOX9 inhibits apoptosis and promotes proliferation, invasion, and metastasis^65–67^. Interestingly, two recent studies^44,45^ have shown that transcriptional upregulation of *Sox9* is an early cellular response to renal injury, and *Sox9* is essential for repair and recovery post AKI. After the initial injury phase, Sox9-expressing renal epithelial cells play a crucial role in the subsequent repair processes. Here we show that renal tubule-specific conditional Sox9-knockout mice are hypersensitive to AKI, indicating that along with its role in recovery and repair, Sox9 plays a pro-survival role in the early phase of AKI.

We also found that Cdkl5 phosphorylates Sox9 at Ser-199 residue during kidney injury in vivo. Cdkl5-mediated phosphorylation seems to reduce the stability of Sox9 protein. Indeed, while the injury-induced transcriptional upregulation of *Sox9* was similar in the control and *Cdkl5*-null mice, *Cdkl5* deletion in RTECs both hastened and markedly increased the accumulation of Sox9 protein (Fig. 4). Pharmacological inhibition of Cdkl5 kinase also resulted in increased accumulation of Sox9 during AKI (Fig. 6). Importantly, examination of the protein stability of various Sox9 mutants (S199A > WT > S199D) indicated that Sox9 phosphorylation at Ser-199 likely causes increased proteasomal degradation resulting in diminished half-life. However, we cannot rule out the possibility that Sox9 phosphorylation at Ser-199 might have other biological effects, including changes in dimerization or altered binding to partner proteins. Ser-199 phosphorylation might also alter the affinity of Sox9 for target genes, a possibility that we cannot currently examine due to the inability to perform ChIP with the phospho-Sox9 antibody. However, these studies have revealed robust Cdkl5-dependent Sox9 phosphorylation in RTECs as part of cellular stress response to distinct injuries.

AKI is associated with a high risk for mortality, development of chronic kidney disease, and multi-organ dysfunction^2,10^. Currently, no specific treatments or prophylactic approaches are available to treat or prevent AKI. We provide proof-of-principle studies showing that targeted Cdkl5 inhibition can provide protection from renal injury. The small-molecule Cdkl5 inhibitor AST-487 mitigated renal injury in multiple mouse models of AKI. While these studies provide promising proof-of-concept data, clinical translation of these studies would depend on the development or identification of Cdkl5 inhibitors with much more specificity than AST-487. Our study also raises three important questions that require further exploration. First, in adults, could systemic Cdkl5 inhibition cause toxicity in the central nervous system? While Cdkl5 is clearly important for early neuronal development, it is unclear if it has any essential function in the adult brain and so, it remains unknown whether short-term pharmacological Cdkl5 inhibition would have any CNS toxicities. However, we propose that the likelihood of any neuronal side effects could be easily reduced by selecting Cdkl5 inhibitors that do not cross the blood–brain barrier. Second, could systemic Cdkl5 inhibition cause toxicity in other peripheral organs or influence renal recovery, regeneration, and fibrosis? Future studies would be required to examine these possibilities; however, we have found that Cdkl5 inhibition not just delays renal injury, but also confers long-term survival benefits, without overt systemic toxicities (Supplementary Fig. 21). Third, it would be critical to examine if Cdkl5 inhibition-dependent Sox9 stabilization has any detrimental long-term effects in the kidneys.

Our study also raises the possibility that the Cdkl5–Sox9 axis might have important biological functions in other nonrenal cell types, especially neurons and cancer cells. An essential question that merits further investigation is whether disruption of CDKL5–SOX9 axis underlies some of the neuronal phenotypes observed in humans and mice with loss-of-function *CDKL5* mutations. Moreover, SOX9 has emerged as an essential regulator of cancer cell stemness, differentiation, and apoptosis. We find that CDKL5 is widely expressed in cancer cell lines (Supplementary Fig. 27), raising the possibility that CDKL5 might regulate SOX9 function in cancer cells. CDKL5 might be a crucial nuclear kinase that suppresses SOX9 function under conditions of cellular stress. Future studies will likely provide insights into these important questions and a better understanding of the biological function of the enigmatic CDKL family of kinases.

## Methods

### Cell culture and reagents

Boston University mouse proximal tubule cells (BUMPT, clone 306, originally from Drs. Wilfred Lieberthal and John Schwartz, Boston University School of Medicine, Boston, MA, and obtained from Dr. Zheng Dong, Augusta University, Augusta, GA) were grown at 37 °C in Dulbecco’s modified Eagle’s medium with 10% fetal bovine serum. The human renal tubular cell line, HK-2 cells (ATCC, CRL-2190) were grown in keratinocyte media (K-SFM) according to the provider’s instructions. Protein kinase inhibitors were obtained from Sigma-Aldrich or Selleckchem. Radiolabeled compounds were obtained from American Radiochemicals or Moravek Biochemicals.

### Primary tubular cell culture and transduction

Anti-GFP antibody and MACS columns (Miltenyi Biotech) were used to isolate GFP-positive tubular epithelial cells. For primary cell culture, tubular epithelial cells were isolated from 6- to 8-week-old male mice^24^. Briefly, mice were euthanized by carbon dioxide asphyxiation, kidneys were excised, and renal cortical tissues were minced thoroughly and digested with 0.75 mg/ml collagenase IV (Thermo Fisher Scientific). RTECs were then purified by centrifugation at 2000 *g* for 10 min in DMEM/F-12 medium with 32% Percoll (Amersham). After washes with serum-free media, the cells were plated in collagen-coated dishes and cultured in DMEM/F-12 medium supplemented with 5 μg/ml transferrin, 5 μg/ml insulin, 0.05 μM hydrocortisone, and 50 μM vitamin C (Sigma-Aldrich). Fresh media was supplemented every alternate day, and after 5–7 days of growth, the isolated proximal tubular cells were trypsinized and replated at 1 × 10^5^ cells per well in 24-well plates. For Cre-mediated gene excision, cultured primary tubular cells were transduced with high-titer (1 × 10^8^ CFU/ml) LV-CMV-Cre-GFP lentivirus (Kerafast), followed by cisplatin treatment 48 h later. Microscopic examinations were carried out to ensure that greater than 90% cells were GFP (Cre) positive before proceeding with cisplatin treatment. For Sox9 “add-back” experiments, proximal tubular cells from WT and Sox9^PT−/−^ cells were transduced with either lentivirus (pLenti-C-Myc-DDK-P2A-Puro, Origene) encoding WT or Sox9 mutants (S199A and S199D). To induce cell death, primary RTECs were incubated with 50 μM cisplatin (Sigma-Aldrich) in fresh culture medium for 24 h, followed by viability and caspase assays.

### siRNA kinome screening

BUMPT cells were used for the siRNA kinome screening using methods similar to our previous study^55^. Briefly, the Dharmacon mouse siRNA library targeting protein kinases and related genes (780 genes) containing four pooled siRNAs for each gene was utilized in the primary screen. Briefly, the BUMPT cells were plated in 96-well plates and reverse-transfected with 25 nM siRNA using Lipofectamine RNAiMAX reagent (Life Technologies). At 48 h post transfection, cells were treated with 15 µM cisplatin in fresh media. Subsequently, 48 h post treatment, CellTiter-Glo luminescent cell viability assay (Promega) was carried out to determine cellular viability. The siRNAs that protected BUMPT cells from cisplatin-induced cell death greater than the positive control (*Pkcδ* siRNA) were selected for secondary screening. The primary screen was carried out in triplicate samples, and data analysis was performed according to established methods^55^.

### Cell viability and caspase assays

Cellular viability was examined using three different assays, namely MTT, CellTiter-Glo, and trypan blue staining. MTT assays were performed using 3-(4,5-dimethylthiazol-2-yl)-2,5-diphenyltetrazolium bromide (MTT) reagent (Sigma-Aldrich). BUMPT cells or RTECs were seeded in 96-well plates, followed by cisplatin treatment for 24–48 h. After treatment, 10 μL of MTT reagent (5 mg/mL MTT in PBS) was added to each well, and plates were incubated at 37 °C with 5% CO_2_ for 4 h, followed by addition of 100 μl of acidified isopropanol (Sigma-Aldrich) and measurement of absorbance at 590 nm. The half-maximal inhibitory concentration (IC_50_) was evaluated by nonlinear regression analysis using GraphPad Prism. Similar to MTT assays, CellTiter-Glo (Promega) assays were performed according to established methods followed by luminescence measurement. Cell viability was also measured by trypan blue exclusion method. Briefly, cells were harvested, followed by trypan blue staining and manual cell counting with a hemocytometer and/or by using Countess Automated Cell Counter (Thermo Fisher); translucent cells were considered as viable and blue-stained cells were counted as dead. Cell viability was calculated by dividing the number of viable cells by the total cell number; each sample was done in triplicate.

Caspase activity was measured in cell lysates using an in vitro assay^68^. Briefly, RTECs were lysed in a buffer containing 1% Triton X-100, and 10 μg of protein from cell lysates was added to an enzymatic assay buffer containing 50 μM DEVD-AFC for 60 min at 37 °C. Fluorescence at excitation 360 nm/emission 535 nm was measured, and free AFC was used to plot a standard curve, and using the standard curve, the fluorescence reading from the enzymatic reaction was converted into the nM AFC liberated per mg protein per hour as a measure of caspase activity.

### Mice breeding

All animals were housed and handled in accordance with approved Institutional Animal Care and Use Committee procedures. All animal studies were conducted according to protocols approved by the Institutional Animal Care and Use Committees of The Ohio State University (2017R00000006). Mice used in the current study were housed in a temperature-controlled environment with a 12-h light cycle and given a standard diet and water ad libitum. Germline *Cdkl5*-deficient mice (stock no. 021967) were obtained from Jackson Laboratories, and heterozygous mice were bred in-house to obtain wild-type and knockout littermates. Conditional gene knockout in RTECs was achieved through breeding of *Cdkl5*-floxed mice (Jackson Laboratory, stock no. 030523) and *Sox9*-floxed mice (Jackson Laboratory, stock no. 013106) with *Ggt1-Cre* mice (Jackson Laboratory, stock no. 012841). Double-knockout mice (dKO^PT^) were generated by crossing Cre-positive Cdkl5 and Sox9-floxed mice. mT/mG mice that express cell membrane-targeted, two-color fluorescent Cre-reporter allele were obtained from Jackson Laboratories (stock no. 007676). In these mice prior to Cre recombination, cell membrane-localized tdTomato (mT) fluorescence expression is widespread in cells/tissues, and Cre recombinase expression induces cell membrane-localized EGFP (mG) fluorescence expression replacing the red fluorescence. The mT/mG mice were bred with *Ggt1-Cre* strain. For all mouse colonies, the pups were ear-tagged and genotyped at 3 weeks of age.

### Animal models of AKI

For all experiments, age-matched (8–12 week) male or female mice were used. Littermates were used in studies with germline, mutant, or conditional knockout mice. For experiments where only wild-type mice were used, 8- to 12-week-old male C57BL/6J or FvB mice were obtained from Jackson Laboratories.

For cisplatin nephrotoxicity experiments, cisplatin (15–30 mg/kg) was administered by i.p. injection^24^. Optimal cisplatin dose was determined for each strain by dose–response experiments. After cisplatin injection, blood was collected on days 0–3 by submandibular vein bleed or on day 3 via cardiac puncture after carbon dioxide asphyxiation. Renal tissues were collected and processed for Western blot and histological analysis.

For ischemia–reperfusion experiments, mice were anesthetized by isoflurane and placed on a surgical platform where the body temperature was monitored throughout the procedure. The skin was disinfected, kidneys were exposed, and bilateral renal pedicles were clamped for 28–35 min. Subsequently, the clamps were released to initiate the reperfusion followed by suturing to close the muscle and skin around the incision. To compensate for the fluid loss, 0.5 ml of warm sterile saline was administered via intraperitoneal injection. Blood was collected on days 0–2 by submandibular vein bleed or on day 2 via cardiac puncture after carbon dioxide asphyxiation. Renal tissues were collected and processed for Western blot and histological analysis. For Cdkl5 pharmacological inhibition studies, vehicle (1:10 v/v N-methylpyrrolidone/PEG300) or AST-487 were administered by oral gavage (25 mg/kg) 6 h post cisplatin injection or ischemic surgery.

To induce rhabdomyolysis, 8–12-week-old male C57BL/6J mice were injected with 7.5 ml/kg 50% glycerol intramuscularly to the two hind legs or injected with saline as a control, followed by blood and tissue collection on day 0–2. To induce FA-mediated kidney injury, male FvB wild-type mice (~25 g, 10 weeks old) were purchased from Jackson Laboratory and administered with FA (250 mg/kg, dissolved in 300 mM NaHCO_3_) through intraperitoneal injection.

### Assessment of renal damage

Renal damage was assessed by serum analysis (blood urea nitrogen and creatinine), histological examination (H&E staining), and analysis of renal expression of injury biomarkers (*Kim1* and Ngal). Mouse blood samples were collected at indicated timepoints, followed by blood urine nitrogen and creatinine measurement by QuantiChrom^TM^ Urea Assay Kit (DIUR-100) and Creatinine Colorimetric Assay Kit (Cayman Chemical). For histological analysis, mouse kidneys were harvested and embedded in paraffin at indicated timepoints before and after AKI induction. Tissue sections (5 µm) were stained with hematoxylin and eosin by standard methods. Histopathologic scoring was conducted in a blinded fashion by examining ten consecutive 100× fields per section from at least three mice per group. Tubular damage was scored by calculation of the percentage of tubules that showed dilation, epithelium flattening, cast formation, loss of brush border and nuclei, and denudation of the basement membrane. The degree of tissue damage was scored based on the percentage of damaged tubules as previously^24^ described: 0: no damage; 1: <25%; 2: 25–50%; 3: 50–75%; 4: >75%.

### Gene expression analysis

Total RNA was extracted from cell lines and murine kidneys using the RNeasy Mini Kit (Qiagen). NanoDrop was used to measure RNA quality and quantity. One microgram of total RNA was then reverse-transcribed using the high-capacity cDNA Reverse Transcription Kit (Thermo Fisher Scientific). qPCR analysis was then performed using the SYBR green master mix with sequence-specific predesigned primers (Sigma). The sequences of qPCR primers are shown in Supplementary Table 2. For quantitative analysis, target gene values were normalized to *β-actin* gene expression using the ΔΔCT value method.

### Protein analysis

Whole-cell lysates from RTECs, cell lines, and renal cortical tissues were made in modified RIPA buffer (20 mM Tris-HCl (pH 7.5), 150 mM NaCl, 1 mM Na_2_EDTA, 1 mM EGTA, 1% NP-40, 2.5 mM sodium pyrophosphate, 1 mM beta-glycerophosphate, protease, and phosphatase inhibitors) supplemented with 1% SDS. Cellular lysates for CDKL5 immunoprecipitation and kinase assay were made in modified RIPA buffer supplemented with 0.1% SDS. For co-immunoprecipitation experiments, cell lysates were made in modified RIPA buffer supplemented with 0.2% β-maltoside. Immunoprecipitations were carried out as described previously^55^ using anti-FLAG (EZview Red ANTI-FLAG M2 Affinity Gel, Sigma-Aldrich), anti-CDKL5 (Millipore, MABS1132), and anti-SOX9 antibodies (Abcam, ab3697). Invitrogen Bis-tris gradient mini or midi-gels were used for western blot analysis, followed by detection by ECL reagent (Cell Signaling). Primary antibodies used for western blot analysis were from Cell Signaling: FLAG (14793), Histone H3 (4499), GAPDH (5174), and Santa Cruz Biotech: β-actin (47778), NGAL (50351), Myoferlin (376879), Sema3e (74554), Gadd45a (6850), Abcam: SOX9 (EPR14335-78), and CDKL5 (ab22453). All primary antibodies were used at 1:1,000 dilution. Secondary antibodies were from Jackson Immunoresearch and used at 1:2,000 dilutions. Uncropped images of western blots are shown in Source Data File. Protein lysates used to determine CDKL5 expression in cancer cell lines were obtained from the DCTD Tumor Repository, National Cancer Institute at Frederick, and the list of cell lines is provided in Supplementary Table 3.

### Protein kinase assay

Protein kinase assays of purified proteins and IP kinases were carried out by in vitro assays^55,68^. For assays with purified proteins, CDKL5-recombinant human protein was obtained from Life Technologies (A30493). To purify Sox9 wild-type and mutant proteins, FLAG-tagged Sox9 constructs were subcloned into pT7CFE1-CHis plasmid (Thermo Fisher). These constructs were then used for in vitro translation using a HeLa cell lysate-based Kit (1-Step Human Coupled IVT Kit—DNA, 88881, Life Technologies). The in vitro-translated proteins were then purified using His Pur cobalt spin columns (Thermo Scientific). For in vitro kinase assays, recombinant CDKL5 and purified Sox9 proteins were incubated in a kinase buffer (Cell Signaling, 9802) supplemented with [gamma-P32] adenosine 5′-triphosphate (ATP) at 30 °C for 30 min. After the incubation period, the reaction was terminated, followed by autoradiographic examination of phosphorylated proteins and subsequent western blot analysis to determine the level of input proteins. For assays used to examine multiple kinase inhibitors, purified kinases (CDK2, CDK4, CDK6, and CDKL5) were incubated with 1 µM concentration of kinase inhibitors for 30 min followed by kinase assays using ADP-Glo Kinase Assay kit (Promega).

Renal tissues and cells were lysed with a buffer containing 150 mM NaCl, 1 mM EDTA, 1 mM EGTA, 1% (vol/vol) Triton X-100, 2.5 mM sodium pyrophosphate, 1 mM β-glycerol phosphate, 1 mM Na_3_VO_4_, 10 μg/ml leupeptin, 10 μg/ml aprotinin, 1 mM phenylmethylsulfonyl fluoride, 50 mM NaF, 0.2% (wt/vol) dodecyl β-d-maltoside, and 20 mM Tris (pH 7.5). The soluble extracts were then subjected to Cdkl5 immunoprecipitation. Briefly, 500 µg of protein lysate was incubated with 2 μg of IgG or anti-Cdkl5 antibody at 4 ^o^C overnight, followed by addition of 30 μl of agarose protein A/G beads. Bead-bound immunoprecipitates were washed and collected by centrifugation. Immunoprecipitates were added to a protein kinase reaction buffer containing 20 µM ATP and myelin basic protein (Millipore) as substrate and incubated at 30 °C for 30 min. The ADP-Glo^™^ Kinase Assay (Promega) kit was then used to measure kinase activity. This is a luminescent ADP detection assay that provides a method to measure kinase activity by quantifying the amount of ADP produced during a kinase reaction. After the reaction was terminated, western blot analysis was carried out to determine the level of inmmunoprecipiated proteins. Relative kinase activity was calculated by normalizing the kinase activity (luminescence) to the amount of IP protein (densitometry of Cdkl5 signal). The specificity of Cdkl5-kinase assay was verified by conducting assays using wild-type and *Cdkl5*^−/y^ tissues, which demonstrated undetectable activity in the *Cdkl5*-deficient tissues (Supplementary Fig. 19a, b).

### Mass spectrometry analysis

Mass spectrometric analysis was performed at the Taplin Biological Mass Spectrometry Facility (Harvard University). Excised gel bands were cut into approximately 1-mm^3^ pieces. Gel pieces were then subjected to a modified in-gel trypsin digestion procedure^69^. Gel pieces were washed and dehydrated with acetonitrile for 10 min, followed by removal of acetonitrile. Pieces were then completely dried in a speed-vac. Rehydration of the gel pieces was with 50 mM ammonium bicarbonate solution containing 12.5 ng/µl modified sequencing-grade trypsin (Promega, Madison, WI) at 4 °C. After 45 min, the excess trypsin solution was removed and replaced with 50 mM ammonium bicarbonate solution to just cover the gel pieces. Samples were then placed in a 37 °C room overnight. Peptides were later extracted by removing the ammonium bicarbonate solution, followed by one wash with a solution containing 50% acetonitrile and 1% formic acid. The extracts were then dried in a speed-vac (~1 h) and reconstituted in 5–10 µl of HPLC solvent A (2.5% acetonitrile and 0.1% formic acid). A nanoscale reverse-phase HPLC capillary column was created by packing 2.6-µm C18 spherical silica beads into a fused silica capillary (100-µm inner diameter × ~30-cm length) with a flame-drawn tip. After equilibrating the column, each sample was loaded via a Famos auto sampler (LC Packings, San Francisco, CA) onto the column. A gradient was formed and peptides were eluted with increasing concentrations of solvent B (97.5% acetonitrile and 0.1% formic acid). As peptides eluted, they were subjected to electrospray ionization and then entered into an LTQ Orbitrap Velos Pro ion-trap mass spectrometer (Thermo Fisher Scientific, Waltham, MA). Peptides were detected, isolated, and fragmented to produce a tandem mass spectrum of specific fragment ions for each peptide. The peptides were fragmented using CID (collision-induced disassociation). A high-resolution scan was done at 60,000 resolutions, followed by 20 low-resolution MS/MS scans in the ion trap. Peptide sequences (and protein identity) were determined by matching protein databases (Uniprot) with the acquired fragmentation pattern by the software program, Sequest Version 3.2 (ThermoFisher, San Jose, CA). The database was indexed based on a trypsin digestion, with two missed cleavages. Fixed modification of 57.0214 Da on cysteine (iodoacetamide) and a variable modification of 15.9949 Da on methionine were considered. The MS1 mass tolerance was 50 ppm and the MS2 tolerance was 1.0 Da. The peptide mass range used was 600–6000 Da. All accepted peptides have a cross-correlation (Xcorr) score of at least 0.5. All databases include a reversed version of all the sequences, and the data were filtered to between a 1 and 2% peptide false-discovery rate. For analysis, we applied a cutoff of five unique peptides per protein. The peptides used for identification of Sox9 are shown in Supplementary Table 4.

### Generation of phospho-Ser-199–SOX9 and phospho-Thr-169-Cdkl5 antibodies

Phospho-specific antibodies were generated and characterized by established methods^70^. Briefly, the rabbit anti-phospho-antibodies were generated by using the 118-day protocol (Covance). Peptide surrounding the Ser-199 of Sox9 and Thr-169 region of Cdkl5 was used for immunization. Immunoblot and ELISA-based methods were used to test the bleeds for antibody production, followed by purification of phospho-antibody by affinity purification. The specificity of the purified antibody was confirmed with in vitro kinase assays and tissues from knockout mice. Dephosphorylation assays were carried out by incubation of cell lysates with recombinant lambda phosphatase (New England Biolabs, P0753) at 30 °C for 2 h, followed by western blot analysis with phospho- and total Sox9 and Cdkl5 antibodies.

### Chromatin immunoprecipitation–qPCR

ChIP assays were performed using the Pierce Magnetic ChIP Kit according to the manufacturer’s instructions^70^. Briefly, cross-linking with 1% formaldehyde was carried out in RTECs or renal tissues, followed by quenching with glycine, cell harvesting, and DNA fragmentation by sonication. Lysates were precleared for 1 h with Protein A + G magnetic beads (EMD Millipore). Precleared lysates were then incubated with 5 μg of anti-SOX9 antibodies (Abcam, ab3697) overnight at 4 °C, followed by addition of Protein A + G magnetic beads and incubation for 4 h at 4 °C. Subsequently, the beads were repeatedly washed, followed by elution of the protein–DNA complexes, reversal of cross-links, and DNA purification. Standard qPCR analysis was then carried out using primers spanning the promoters of target genes. The sequences of primers are shown in Supplementary Table 2.

### Plasmids and site-directed mutagenesis

The *Cdkl5* and *Sox9* plasmids with pCMV6-entry backbone were obtained from Origene. The QuikChange II XL Site-Directed Mutagenesis Kit (Agilent) was utilized to generate mutants, according to suggested methods. The QuikChange primer design program was employed to design mutagenesis primers^55^. Primers were synthesized by Integrated DNA Technologies. All constructs were sequenced to confirm successful mutagenesis. The mutagenesis primer sequences are shown in Supplementary Table 2.

### Promoter luciferase assay

HEK293 cells were stably transfected with either empty vector (pCMV6) or Sox9 expression vector (Origene). These cells were then utilized for promoter luciferase reporter assays^70^. Briefly, 5 × 10^3^ cells were plated overnight on white poly-l-lysine-coated 96-well plates, followed by transient transfection with either promoter constructs (Switchgear Genomics, encoding 2-kb sequence upstream of transcription start sites of the following genes: Gadd45a, Wwp2, Sema3e, and Myof) or empty promoter construct at 30 ng in combination with the Cypridina TK control construct (Switchgear Genomics) at 1 ng, according to the manufacturer’s protocol (Switchgear Genomics, Lightswitch Dual Assay kit, DA010). The promoter construct encodes a Renilla luminescent reporter gene, called RenSP, while the transfection and normalization vector encodes a Cypridina luciferase. The Renilla luciferase activity was normalized with the Cypridina luciferase activity.

### Statistical considerations

Data are presented as mean with s.e.m., unless stated otherwise. Statistical calculations (Student’s *t* test or analysis of variance) were carried our using GraphPad Prism. *p* < 0.05 was considered statistically significant. To calculate statistical significance between two groups, two-tailed unpaired Student’s *t* test was performed. One-way ANOVA followed by Tukey’s or Dunnett’s multiple-comparison test was used for comparisons among three or more groups. For all the experimental data presented in the paper, no sample outliers were excluded.

### Reporting summary

Further information on research design is available in the Nature Research Reporting Summary linked to this article.

## Supplementary information

Supplementary Information

Reporting Summary

## Data Availability

The source data underlying Figs. 1b, 1d, 1e–h, 2a–f, 2h–k, 2n, 3a–k, 4a, b, 4d–f, 5a–k, 6a–I, and 7a–g and Supplementary Figs. 1a–k, 2a, b, 4a–i, 5a–f, 6a–I, 7, 8a–c, 9a, b, 10a–d, aa, 12a–e, 13a–e, 14a–h, 15a–h, 16, 17a–p, 18a–h, 19a–e, 20, 21a, b, 22a–f, 23a–g, 24a–g, 25a–g, 26a–c, and 27 are provided as a Source Data file. A reporting summary for this article is available as a Supplementary Information file. All data supporting the findings of this study are available from the corresponding author on reasonable request.

## Source Data

Source Data
