# Supplementary material for: A kinome-wide screen identifies a CDKL5-SOX9 regulatory axis in epithelial cell death and kidney injury
Source: Nat Commun. 2020 Apr 21;11:1924. doi: 10.1038/s41467-020-15638-6 (PMC7174303; doi:10.1038/s41467-020-15638-6)
Supplement: Supplementary file 1 — Supplementary Information [file 41467_2020_15638_MOESM1_ESM.pdf]

Supplementary Information for

'***A Kinome-wide screen identifies a CDKL5-SOX9 regulatory axis in epithelial cell death and kidney injury***' **by** ***Kim J.Y. et.al***

Supplementary Figures (1-27)

Supplementary Tables (1-4)

Supplementary Figure 1

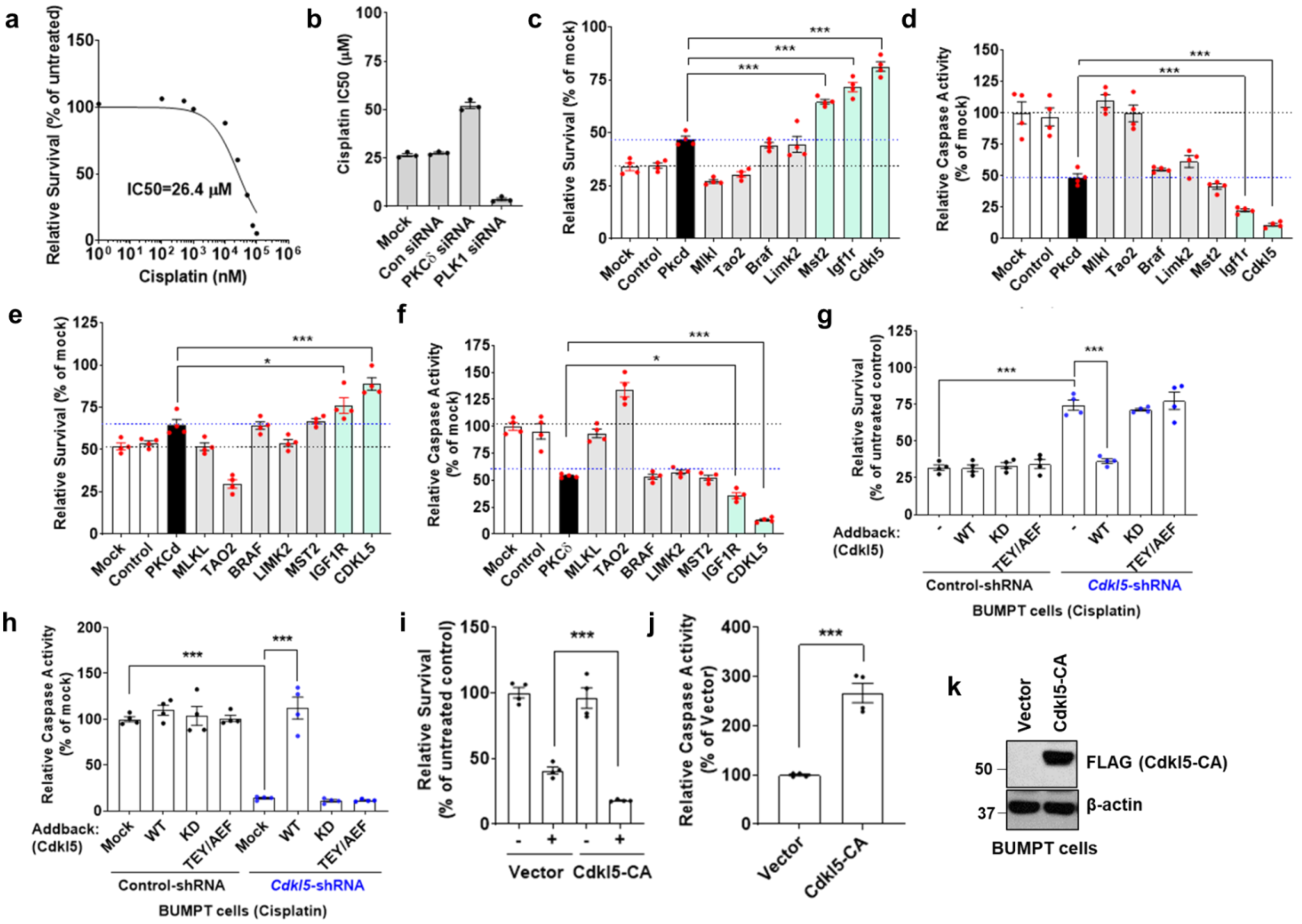

Supplementary Figure 1: Primary and confirmatory RNAi screens identify CDKL5 kinase as a novel regulator of renal epithelial cell death. (**a**) BUMPT cells, a murine renal tubular cell line was treated with cisplatin at indicated doses followed by cellular viability assay (MTT). Data is representative of four independent experiments. (**b**) BUMPT cells were transfected with indicated siRNAs, followed by dose response experiments (MTT) to determine cisplatin IC50. Data are presented as individual data points (n = 3 biologically independent samples), from one out of two independent experiments, both producing similar results (**c-d**) BUMPT cells were transfected with indicated siRNAs followed by cisplatin treatment and measurement of cellular viability by trypan blue staining and measurement of caspase activity in cell lysates. Data are presented as individual data points (n = 4 biologically independent samples), from one out of three independent experiments, all producing similar results. (**e-f**) HK-2 cells, a human renal tubular cell line was transfected with indicated siRNAs followed by cisplatin treatment and measurement of cellular viability by trypan blue staining and measurement of caspase activity in cell lysates. Data are presented as individual data points (n = 4 biologically independent samples), from one out of two independent experiments, both producing similar results. (**g-h**) BUMPT cells were stably transfected with indicated shRNAs followed transient transfection with indicated addback plasmids, followed by cisplatin treatment and measurement of cellular viability by trypan blue staining and measurement of caspase activity in cell lysates. Data are presented as individual data points (n = 4 biologically independent samples), from one out of three independent experiments, all producing similar results. (**i-k**) BUMPT cells were transiently transfected with either empty vector or plasmid encoding catalytically active Cdkl5 (denoted as Cdkl5-CA, NT kinase domain) followed by treatment with either vehicle (-) or cisplatin (+). Trypan blue based viability assays showed that Cdkl5-CA can sensitizes RTECs to cisplatin-associated cell death. Caspase assays of cisplatin treated cells also showed increased caspase activity in Cdkl5-CA transfected cells. The representative western blot shows the expression of FLAG tagged Cdkl5-CA in BUMPT cells. Data (i-j) are presented as individual data points (n = 4 biologically independent samples), from one out of two independent experiments, both producing similar results. In all the bar graphs, experimental values are presented as mean ± s.e.m. The height of error bar=1 s.e. and p<0.05 was indicated as statistically significant. Student's t-test (j) or 1-way ANOVA followed by Dunnett's (c-f) or Tukey's multiple-comparisons test (g-i) were carried out and statistical significance is indicated by *p < 0.05, **p < 0.01, ***p < 0.001. Source data are provided as a Source Data file.

Supplementary Figure 2

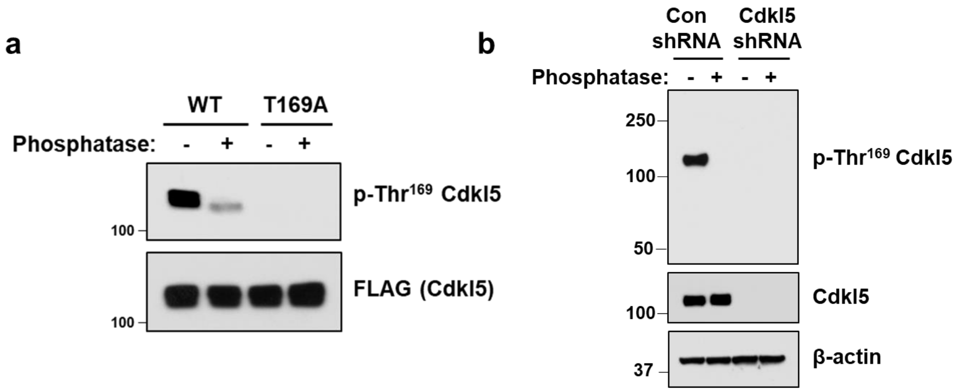

Supplementary Figure 2: Characterization of phospho-Thr-169 Cdkl5 antibody. (**a**) BUMPT cells were transiently transfected with FLAG-tagged WT or T169A Cdkl5 plasmids and 24 hours later, lysates were collected, followed by Cdkl5 immunoprecipitation using anti-FLAG beads. Immunoprecipitates were either left untreated (-) or phosphatase treatment (+) was carried out for 3 hours. Subsequently western blot analysis was carried out with phospho-Cdkl5 and FLAG antibodies. Blot is representative of two independent experiments. (**b**) Cellular lysates from BUMPT cells stably transfected with indicated shRNAs and treated with cisplatin for 6 hours were used for western blot analysis of total and phospho-Cdkl5 protein levels. Blots are representative of two independent experiments. Source data are provided as a Source Data file.

Supplementary Figure 3

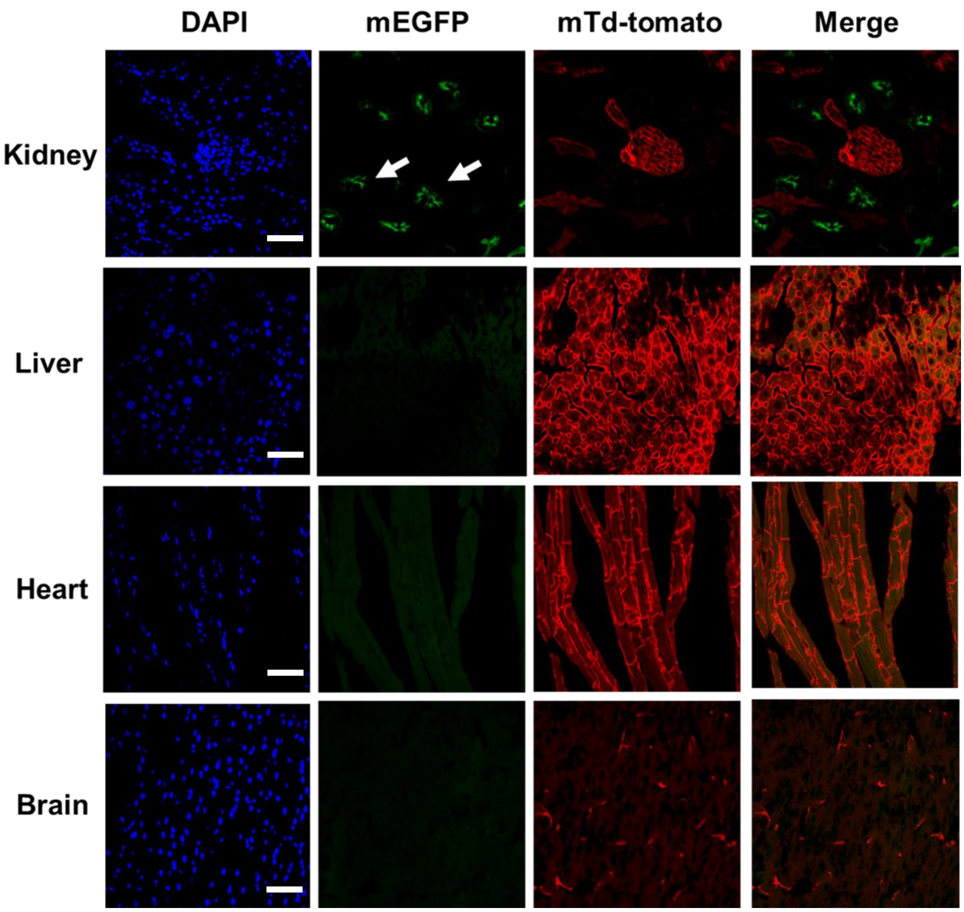

Supplementary Figure 3: Characterization of *Ggt1-Cre* mice. The ROSA mT/mG mice were crossed with the renal tubular epithelial cell-specific *Ggt1-Cre* (gamma-glutamyltransferase 1) mice to generate transgenic mice that express membrane-localized GFP in the tubular epithelial cells. Before Cre-mediated recombination, all cell types express membrane localized Td tomato. Indicated tissues from Cre positive mT/mG mice were examined for Cre-mediated expression of EGFP. Representative image showing EGFP expression in renal tubules (arrow), while other tissues did not express EGFP. Images were taken with confocal microscope at 60X magnification. Scale bar: 100 µm.

Supplementary Figure 4

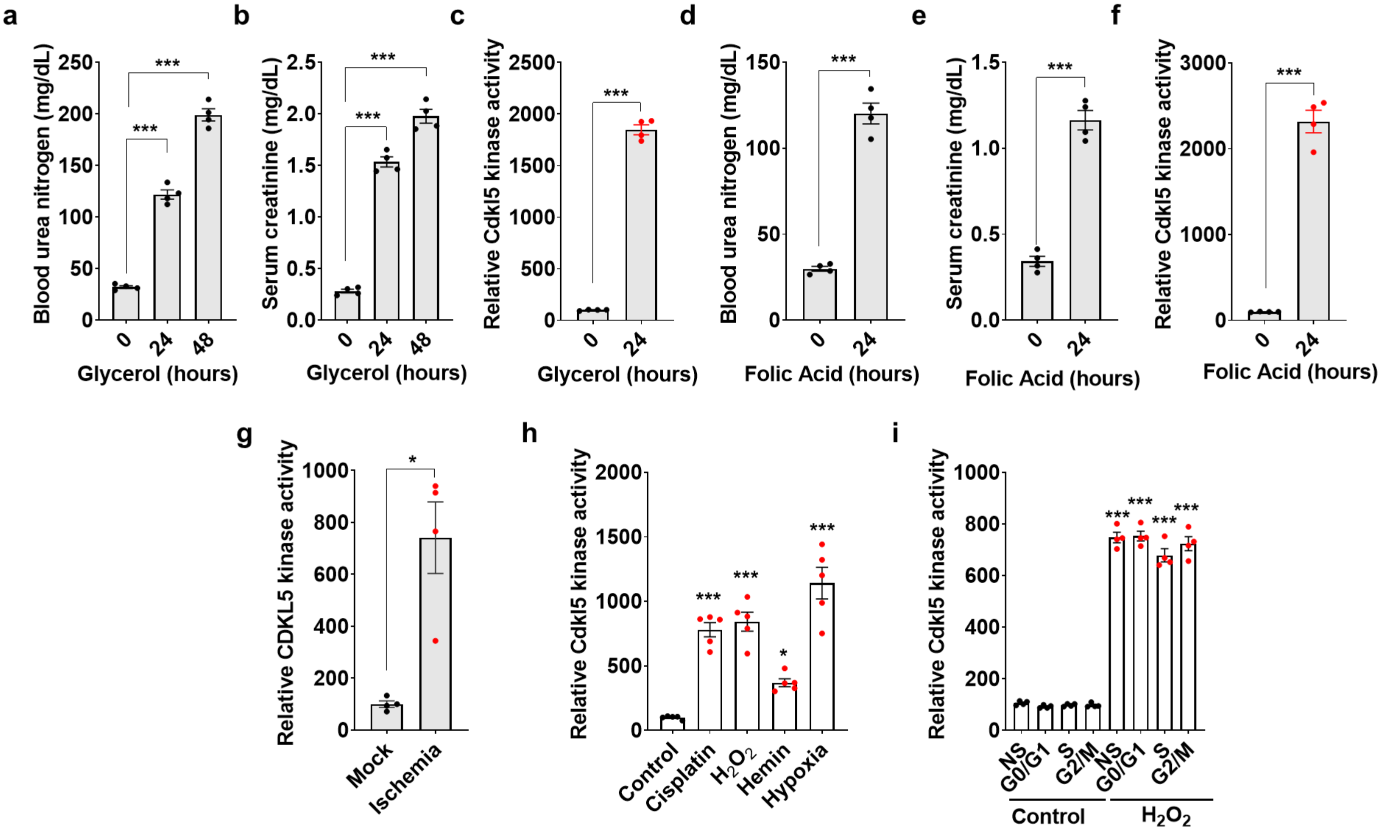

Supplementary Figure 4: Stress responsive and cell cycle independent activation of CDKL5. (**a-c**) Rhabdomyolysis was induced in male wild-type (C57BL/6) mice by glycerol injection (7.5 ml/kg 50% glycerol) in the hind-leg muscles followed by measurement of renal function (BUN and Creatinine) and renal Cdkl5 activity (Kinase assay). Data are presented as individual data points (n = 4 biologically independent samples), from one out of two independent experiments, all producing similar results. (**d-f**) Male wild-type (FvB) mice were injected with folic acid (250 mg/kg, i.p.) followed by measurement of renal function (BUN and Creatinine) and renal CDKL5 activity (Kinase assay). Data are presented as individual data points (n = 4 biologically independent samples), from one out of two independent experiments, both producing similar results. (**g**) Renal tissues from a previously described porcine model of ischemic acute kidney injury were subjected to CDKL5 kinase assay. Data are presented as individual data points (n = 4 biologically independent samples), from one out of two repeat experiments, both producing similar results. (**h**) Cellular stress was induced in primary tubular cells by cisplatin, hydrogen peroxide, hemin or 1% hypoxia treatment followed by Cdkl5 kinase assays. Data are presented as individual data points (n = 5 biologically independent samples), from one out of three independent experiments, all producing similar results. (**i**) primary tubular cells were synchronized by double thymidine block and then released into nocodazole-containing medium. Non-synchronized and synchronized cells were then treated with hydrogen peroxide for 1 hour, followed by assessment of CDKL5 kinase activity. Data are presented as individual data points (n = 3 biologically independent samples), from one out of two independent experiments, all producing similar results. In all the bar graphs, experimental values are presented as mean ± s.e.m. The height of error bar=1 s.e. and p<0.05 was indicated as statistically significant. 1-way ANOVA followed by Dunnett's (a,b, h, and i) or students t-test (c,d,e, f, and g) was carried out and statistical significance is indicated by *p < 0.05, **p < 0.01, ***p < 0.001. Source data are provided as a Source Data file.

Supplementary Figure 5

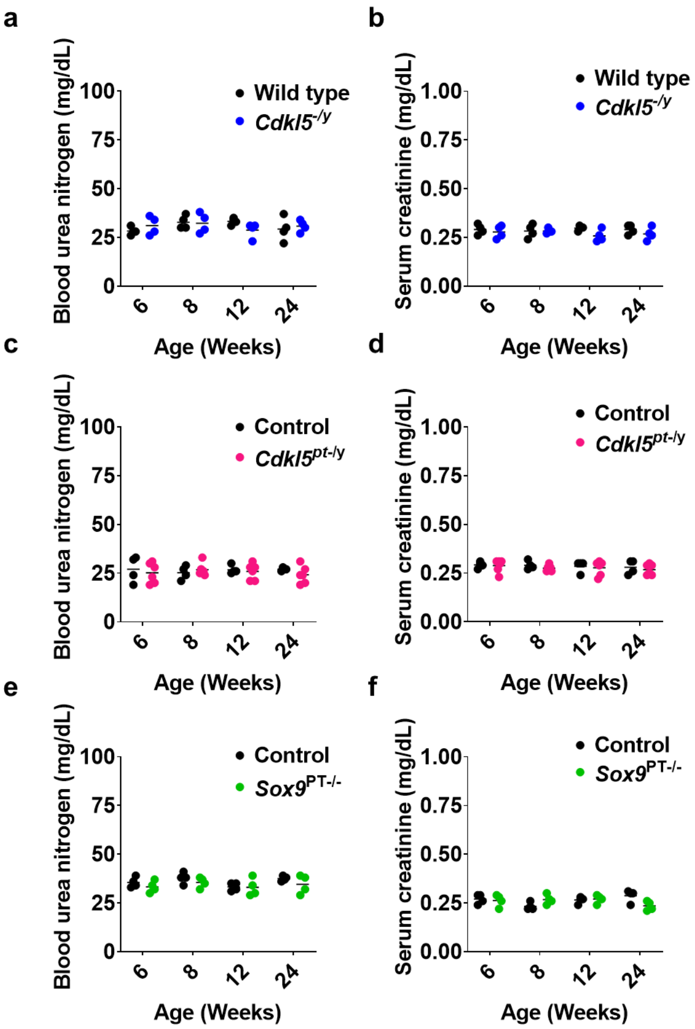

Supplementary Figure 5: Effect of gene knockout on renal function. Renal function (BUN and Creatinine) was examined in littermates with indicated genotypes from 6-24 weeks of age under baseline conditions. These results show that germline Cdkl5 knockout (**a-b**), renal tubule specific Cdkl5 knockout (**d-e**) or conditional Sox9 gene knockout (**g-h**) does not affect kidney function under normal conditions. Data are presented as individual data points (n = 4 biologically independent samples), from a single long-term experiment. Source data are provided as a Source Data file.

Supplementary Figure 6

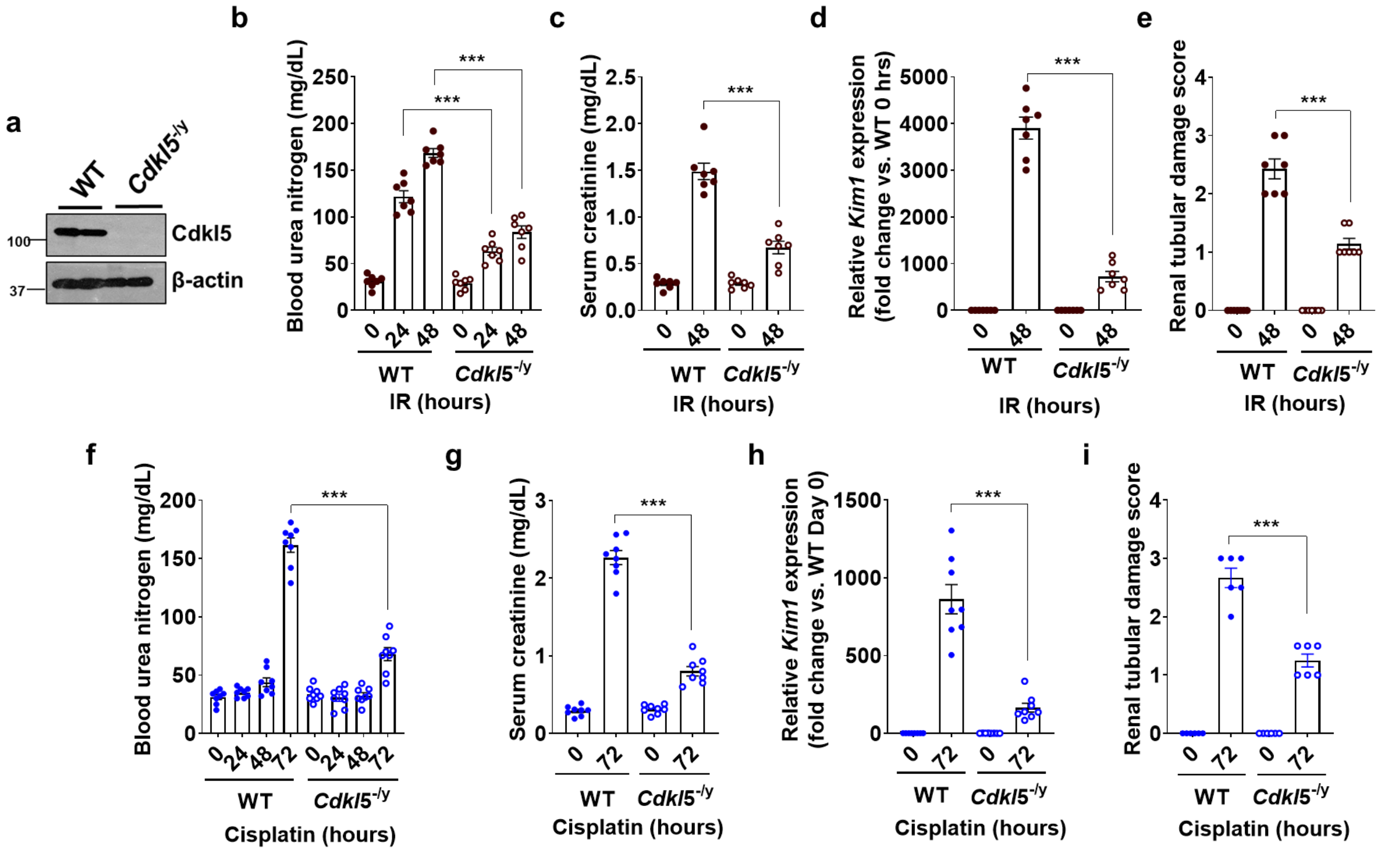

Supplementary Figure 6: Ischemic and nephrotoxic AKI is mitigated in *Cdkl5* knockout mice. (**a**) Representative western blot confirming Cdkl5 gene ablation in renal tissues. Bilateral renal ischemia was induced in wild-type and *Cdkl5*$^{-/y}$ mice for 28 minutes followed by examination of renal function and damage post-reperfusion. (**b**) Blood urea nitrogen (**c**) Serum creatinine (**d**) renal *Kim1* mRNA expression (**e**) renal histological analysis (H&E) showed that *Cdkl5* deficiency confers protection from ischemia-associated AKI. Data presented (b-e) is cumulative of three independent experiment (n=7). Wild-type and *Cdkl5*$^{-/y}$ mice were treated with cisplatin (25 mg/kg) followed by examination of renal function. (**f**) Blood urea nitrogen (**g**) Serum creatinine (**h**) renal *Kim1* mRNA expression (**i**) renal histological analysis (H&E) showed that *Cdkl5* contributes to cisplatin-mediated AKI. Data presented (f-i) is cumulative of two out of four independent experiment (n=8), that showed similar results. In all the bar graphs, experimental values are presented as mean ± s.e.m. The height of error bar=1 s.e. and p<0.05 was indicated as statistically significant. 1-way ANOVA followed by Tukey's multiple-comparisons test was carried out and statistical significance is indicated by *p < 0.05, **p < 0.01, ***p < 0.001. Source data are provided as a Source Data file.

Supplementary Figure 7

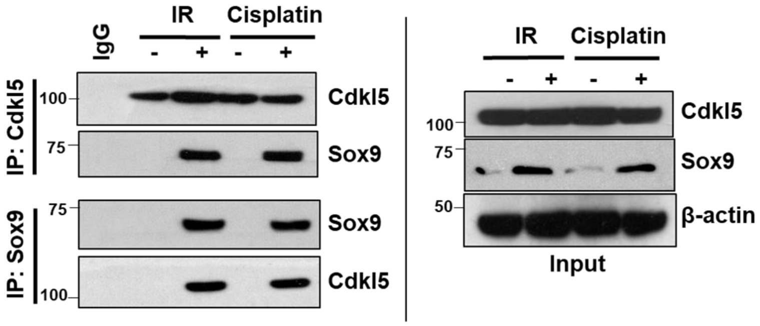

Supplementary Figure 7: Cdkl5 interacts with Sox9. Renal tissues from control (-) and either ischemia (+) or cisplatin (+) treated mice were used for Cdkl5 or Sox9 immunoprecipitation followed by western blot analysis of indicated proteins. IgG group was used as negative control. Blots are representative of three independent experiments. These reciprocal immunoprecipitation experiments show that Cdkl5 interacts with Sox9 during AKI. Source data are provided as a Source Data file.

Supplementary Figure 8

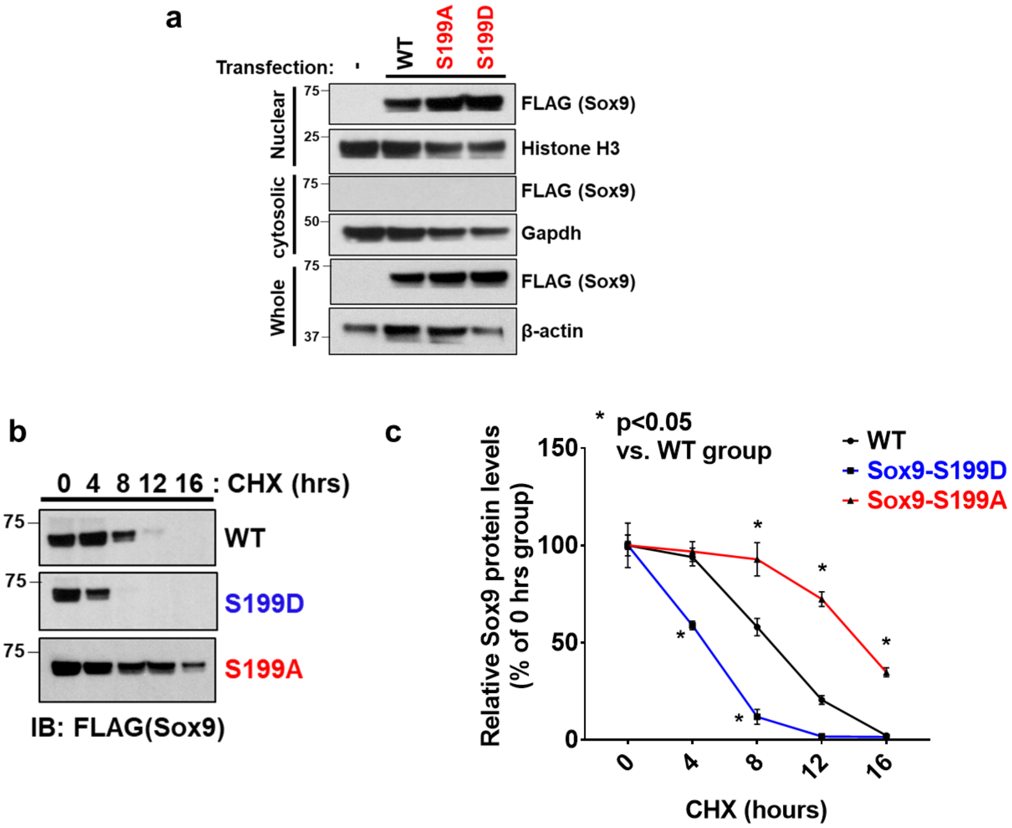

Supplementary Figure 8: Characterization of Sox9 S199 mutants. (a) BUMPT cells were transiently transfected with empty vector (-), or FLAG tagged WT as well as S199A and S199D Sox9 plasmids. One day later, cellular fractionation was carried out using Thermo Scientific Subcellular Protein Fractionation Kit. Lysates from various fractions were then subjected to western blot analysis of indicated proteins. Blot is representative of two independent experiment. (**b-c**) BUMPT cells were transiently transfected with FLAG tagged wild-type or S199A (non-phosphorylatable) and S199D (phospho-mimetic) Sox9 mutant constructs. One day later, cells were incubated with or without cycloheximide (100 µg/ml) for the indicated times, followed by western blot analysis of FLAG-tagged Sox9. Densitometric analysis was then performed to examine Sox9 protein stability. Graph represents data (n=3 biologically independent samples) from one out of three independent experiment. *indicates $p<0.05$ as compared to the WT group. Source data are provided as a Source Data file.

Supplementary Figure 9

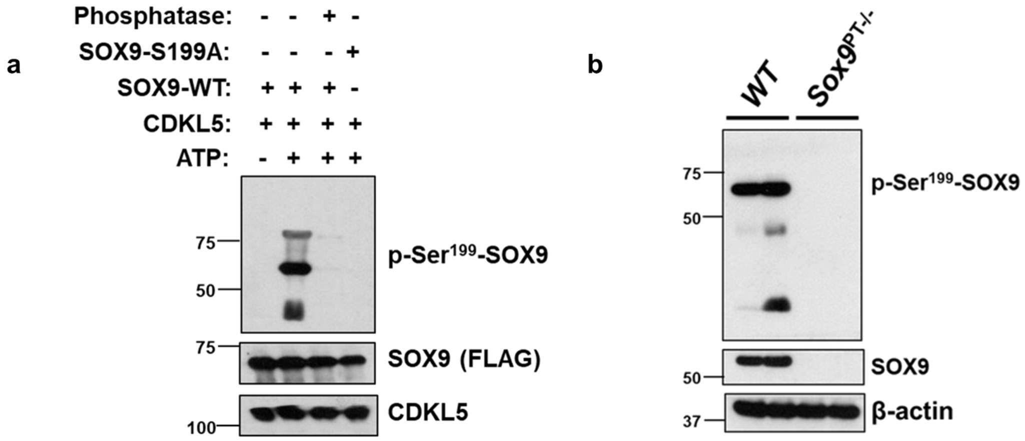

Supplementary Figure 9: Validation of phospho-Ser-199 Sox9 antibody. (**a**) *In vitro* kinase assays were carried out with cold ATP using recombinant CDKL5-kinase and wild-type or SOX9-S199A mutant proteins as substrates followed by western blot analysis of indicated proteins. In one group phosphatase treatment was carried out for 3 hours. Blot is representative of two independent experiment. (**b**) Ischemic renal tissues from control and *Sox9*$^{PT-/-}$ mice were subjected to immunoblot analysis of indicated proteins. Blots are representative of two independent experiments. Source data are provided as a Source Data file.

Supplementary Figure 10

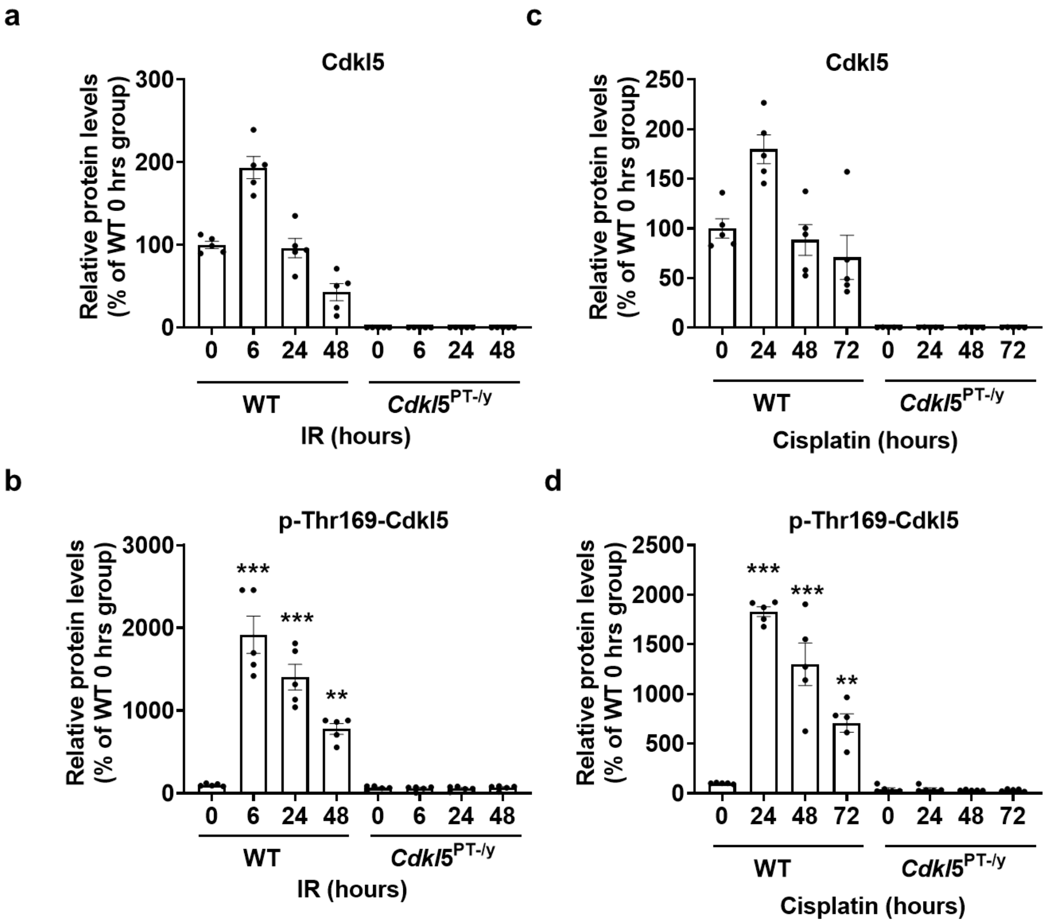

Supplementary Figure 10: Increased Cdkl5 phosphorylation in the TEY motif during AKI. (**a-b**) Littermate control and *Cdkl5* conditional knockout mice (*Cdkl5*$^{PT-/y}$) were challenged with bilateral renal ischemia followed by western blot analysis of total and phospho-Thr-169 Cdkl5 protein in the renal cortical tissues as shown in the representative blots in Figure 4e. The graphs represent densitometric analysis (normalized to β-actin levels) of five distinct samples from three independent experiments. (**c-d**) Control and Cdkl5$^{PT-/y}$ male mice were challenged with cisplatin injection, followed by western blot analysis of total and phospho-Thr-169 Cdkl5 protein in the renal cortex as shown in the representative blots in Figure 4d. The graphs represent densitometric analysis (normalized to β-actin levels) of five distinct samples from three independent experiments. No positive signals were detected by both the total and phospho-Thr-169 antibody in the *Cdkl5*$^{PT-/y}$ tissues, confirming the antibody specificity. In all the bar graphs, experimental values are presented as mean ± s.e.m. The height of error bar=1 s.e. and $p<0.05$ was indicated as statistically significant. 1-way ANOVA followed by Dunnett's multiple-comparisons test (b-d, vs. WT 0hrs. group) was carried out and statistical significance is indicated by *$p < 0.05$, **$p < 0.01$, ***$p < 0.001$. Source data are provided as a Source Data file.

Supplementary Figure 11

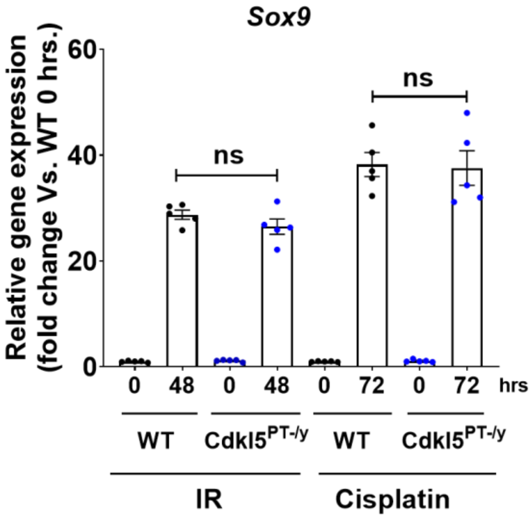

Supplementary Figure 11: Cdkl5 gene ablation does not influence AKI-induced Sox9 mRNA upregulation. qPCR analysis of *Sox9* mRNA was carried in the renal cortical tissues of control and mice with renal injury at the indicated time-points. AKI-induced *Sox9* mRNA up-regulation was not altered by *Cdkl5* deficiency. Graph represents data (n=4 biologically independent samples) from one out of two independent experiments. In the bar graph, experimental values are presented as mean ± s.e.m. The height of error bar=1 s.e. and $p<0.05$ was considered as statistically significant, while ns indicates not significant. 1-way ANOVA followed by Tukey's multiple-comparisons test was carried out and statistical significance is indicated by *$p < 0.05$, **$p < 0.01$, ***$p < 0.001$. Source data are provided as a Source Data file.

Supplementary Figure 12

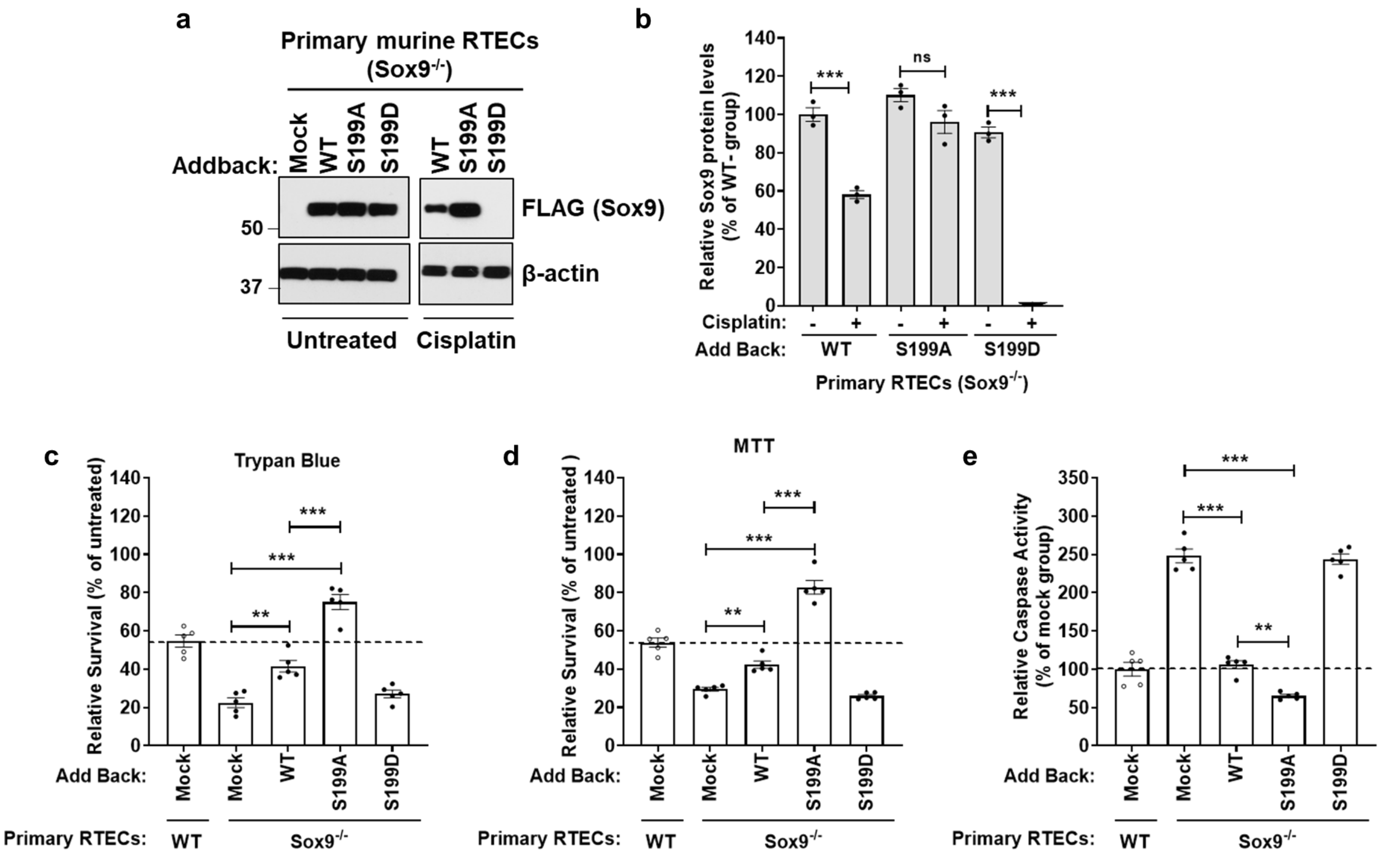

Supplementary Figure 12: Effect of Sox9 mutations at S199 site on RTEC survival. (a-b) Primary murine RTECs were isolated from Sox9 conditional knockout mice. After culturing for one week, lentiviral mediated transduction was carried out with empty vector (mock) or FLAG-tagged WT or mutant Sox9 (S199A and S199D). Two days after transduction, cells were treated with 50 μM Cisplatin for 24 hours followed by western blot analysis of FLAG tagged Sox9. The blots are representative of three independent experiments. The graph depicts densitometric analysis of Sox9 protein levels (FLAG). (**c-e**) Primary murine RTECs were isolated from WT and Sox9 conditional knockout mice. After culturing for one week, lentiviral mediated transduction was carried out with empty vector (mock) or FLAG-tagged WT or mutant Sox9 (S199A and S199D). Two days after transduction, cells were treated with 50 μM Cisplatin for 24 hours followed by cell viability assessment and caspase assays using cellular lysates. Graph represents data (n=5 biologically independent samples) from one out of three independent experiments. In the bar graph, experimental values are presented as mean ± s.e.m. The height of error bar=1 s.e. and $p<0.05$ was considered as statistically significant, while ns indicates not significant. 1-way ANOVA followed by Tukey's multiple-comparisons test was carried out and statistical significance is indicated by *$p < 0.05$, **$p < 0.01$, ***$p < 0.001$. Source data are provided as a Source Data file.

Supplementary Figure 13

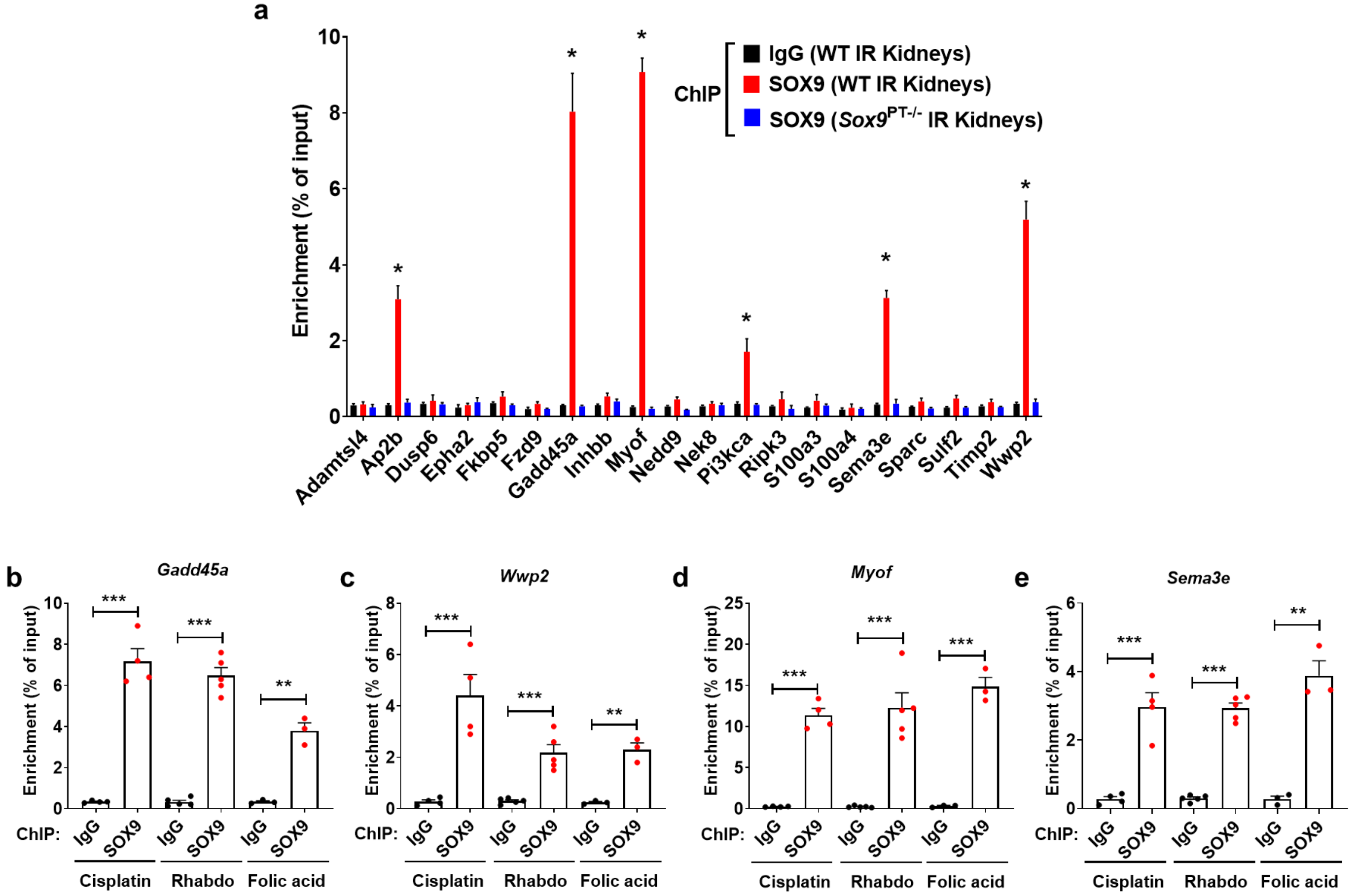

Supplementary Figure 13: Identification of Sox9 target genes in renal tubular cells. (**a**) Sox9 Chromatin immunoprecipitations (ChIP) were carried out in indicated ischemic renal tissues followed by qPCR analysis to determine target gene binding. Data are shown as mean of 3 replicates and representative of two independent experiments. (**b-e**) Sox9 binding to putative target gene (*Gadd45a, Wwp2, Myof* and *Sema3e)* promoter regions was assesed in three distinct models of AKI, namely, cisplatin nephrotoxicity, rhabdomyolysis and folic acid-associated AKI. Data are presented as individual data points (n = 3-4 biologically independent samples), from one out of three independent experiments, all producing similar results. In the bar graph, experimental values are presented as mean ± s.e.m. The height of error bar=1 s.e. and p<0.05 was considered as statistically significant, while ns indicates not significant. 1-way ANOVA followed by Dunnett's multiple-comparisons test was carried out and statistical significance is indicated by *p < 0.05, **p < 0.01, ***p < 0.001. Source data are provided as a Source Data file.

Supplementary Figure 14

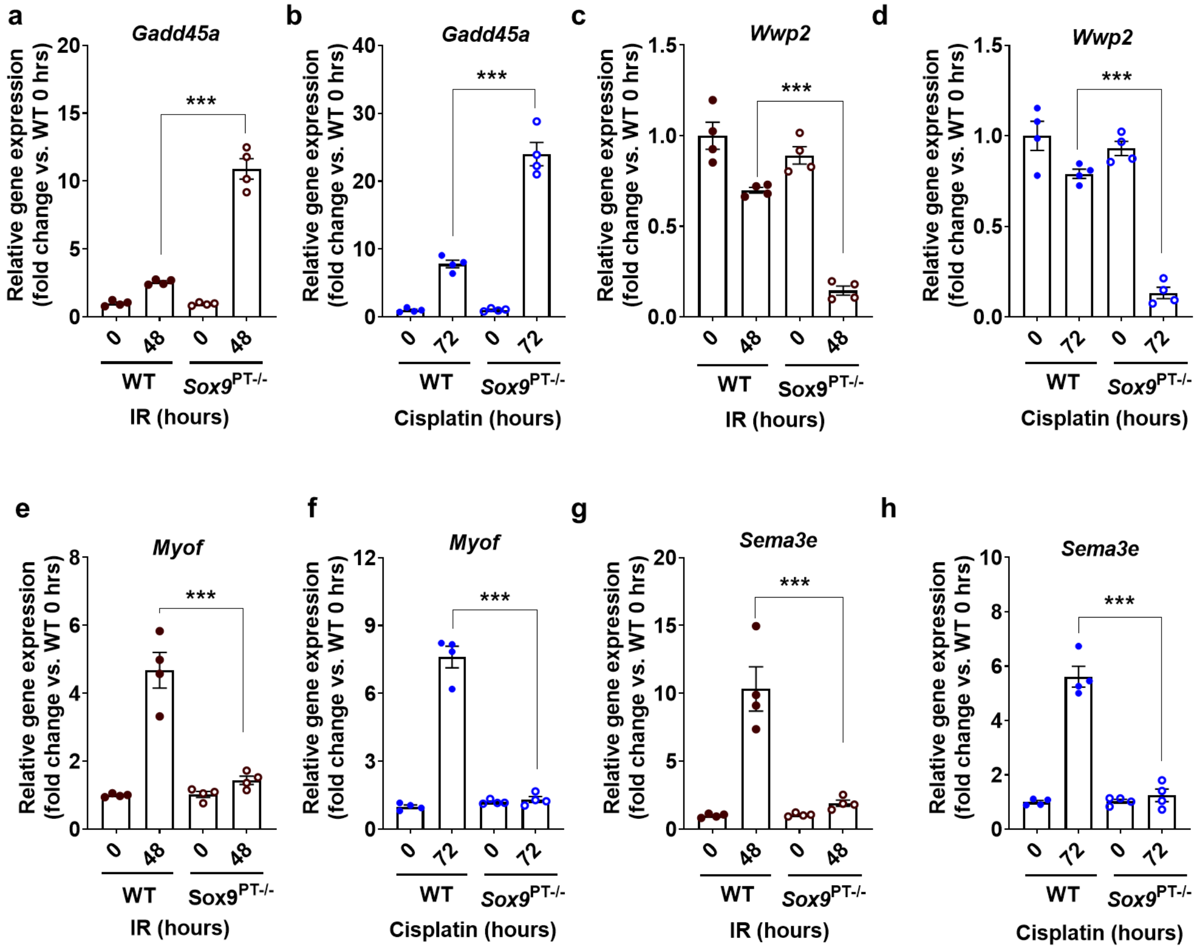

Supplementary Figure 14: Gene expression analysis of putative Sox9 target genes in Sox9 conditional knockout mice. Bilateral renal ischemia was induced in wild-type and *Sox9*$^{PT-/-}$ mice for 30 minutes or wild-type and *Sox9*$^{PT-/-}$ mice were treated with cisplatin (30 mg/kg) followed by qPCR based analysis of gene expression in renal cortical tissues. These results indicate that Sox9 potentially suppresses Gadd45a expression (**a-b**) during AKI. On the other hand Sox9 seems to be essential for upregulation of Wwp2 (**c-d**), Myof (**e-f**) and Sema3e (**g-h**) during AKI. Data are presented as individual data points (n = 4 biologically independent samples), from one out of two independent experiments, all producing similar results. In the bar graph, experimental values are presented as mean ± s.e.m. The height of error bar=1 s.e. and $p<0.05$ was considered as statistically significant, while ns indicates not significant. 1-way ANOVA followed by Tukey's multiple-comparisons test was carried out and statistical significance is indicated by *$p < 0.05$, **$p < 0.01$, ***$p < 0.001$. Source data are provided as a Source Data file.

Supplementary Figure 15

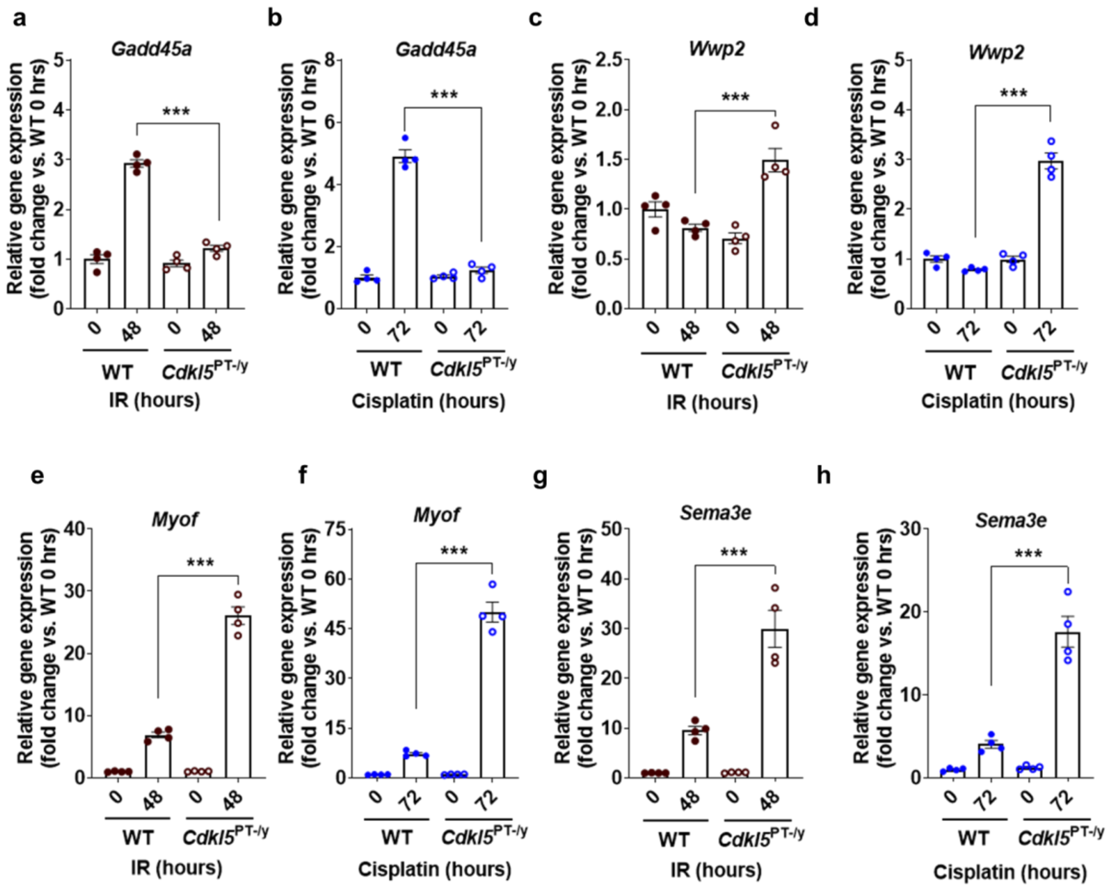

Supplementary Figure 15: Effect of Cdkl5 deficiency on the expression of putative Sox9 target genes. Ischemic or nephrotoxic AKI was induced in wild-type and *Cdkl5*$^{PT-/-}$ mice followed by qPCR based analysis of gene expression in renal cortical tissues. These results indicate that Cdkl5 deficiency leads to suppression of Gadd45a expression (**a-b**) during AKI. On the other hand Cdkl5 deficiency leads to increased upregulation of Wwp2 (**c-d**), Myof (**e-f**) and Sema3e (**g-h**) during AKI. Data are presented as individual data points (n = 4 biologically independent samples), from one out of two independent experiments, all producing similar results. In the bar graph, experimental values are presented as mean ± s.e.m. The height of error bar=1 s.e. and $p<0.05$ was considered as statistically significant, while ns indicates not significant. 1-way ANOVA followed by Tukey's multiple-comparisons test was carried out and statistical significance is indicated by *$p < 0.05$, **$p < 0.01$, ***$p < 0.001$. Source data are provided as a Source Data file.

Supplementary Figure 16

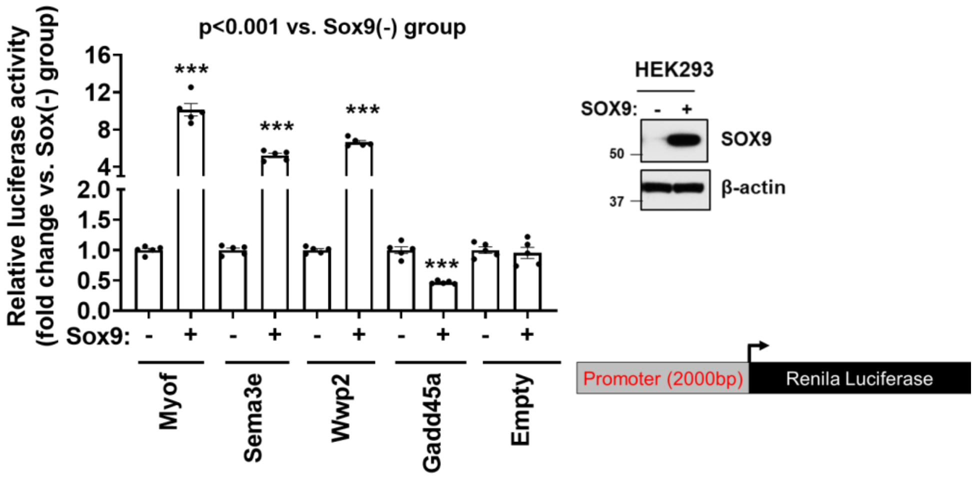

Supplementary Figure 16: Reporter assays for detection of SOX9 binding at putative target gene promoters. HEK293 cells, which express low levels of endogenous SOX9 protein were stably transfected with empty plasmid or SOX9 construct. The mock transfected cells (-) and SOX9 expressing cells (+) were then used for luciferase based reporter assays. Promoter sequences (-2000bp from TSS) for the four target genes were cloned in a luciferase reporter construct. Mock (-) and SOX9 (+) expressing HEK293 cells were transiently co-transfected with reporter renila luciferase contructs (empty, Myof, Sema3e, Wwp2, and Gadd45a) and reference cypridina luciferase (normalizing control), followed by measurement of luciferase activity at 24 hours. The normalized luciferase activity of mock (-) group was then compared with the SOX9 (+) expression group for each target gene. Data in the graphs are presented as individual data points (n = 5 biologically independent samples), from one out of three independent experiments, all producing similar results. In the bar graph, experimental values are presented as mean ± s.e.m. The height of error bar=1 s.e. and $p<0.05$ was considered as statistically significant. Student's *t* test was used and statistical significance is indicated by *$p < 0.05$, **$p < 0.01$, ***$p < 0.001$. Source data are provided as a Source Data file.

Supplementary Figure 17

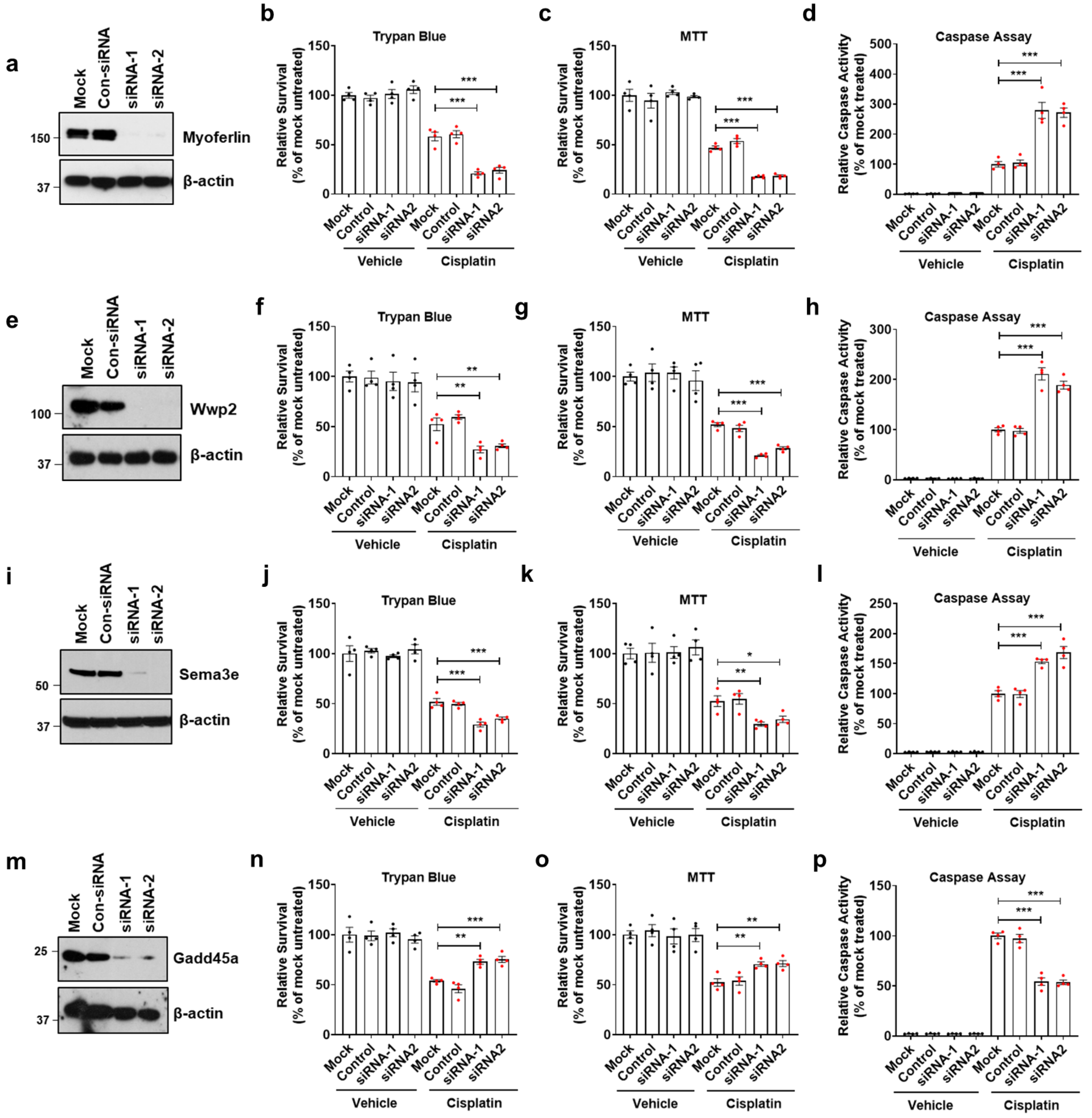

Supplementary Figure 17: Functional role of putative Sox9 target genes in renal epithelial cell death. BUMPT cells were transfected with indicated siRNAs (one control non-specific siRNA and two distinct siRNAs targeting the same gene, indicated as siRNA1 and 2) followed by cisplatin treatment for 24 hours and subsequent assessment of cell viability and caspase activity. Myoferlin (**a-d**), Wwp2 (**e-h**), and Sema3e (**i-l**) knockdown sensitizes BUMPT cells to cisplatin associated cell death. On the other hand Gadd45a knockdown (**m-p**) provided protection from cisplatin mediated cell death. Western blots (a, e, I, and m) of siRNA transfected and untreated cells are representative of two independent experiments. Data in the graphs are presented as individual data points (n = 4 biologically independent samples), from one out of three independent experiments, all producing similar results. In the bar graph, experimental values are presented as mean ± s.e.m. The height of error bar=1 s.e. and $p<0.05$ was considered as statistically significant. 1-way ANOVA followed by Tukey's multiple-comparisons test was carried out and statistical significance is indicated by *$p < 0.05$, **$p < 0.01$, ***$p < 0.001$. Source data are provided as a Source Data file.

Supplementary Figure 18

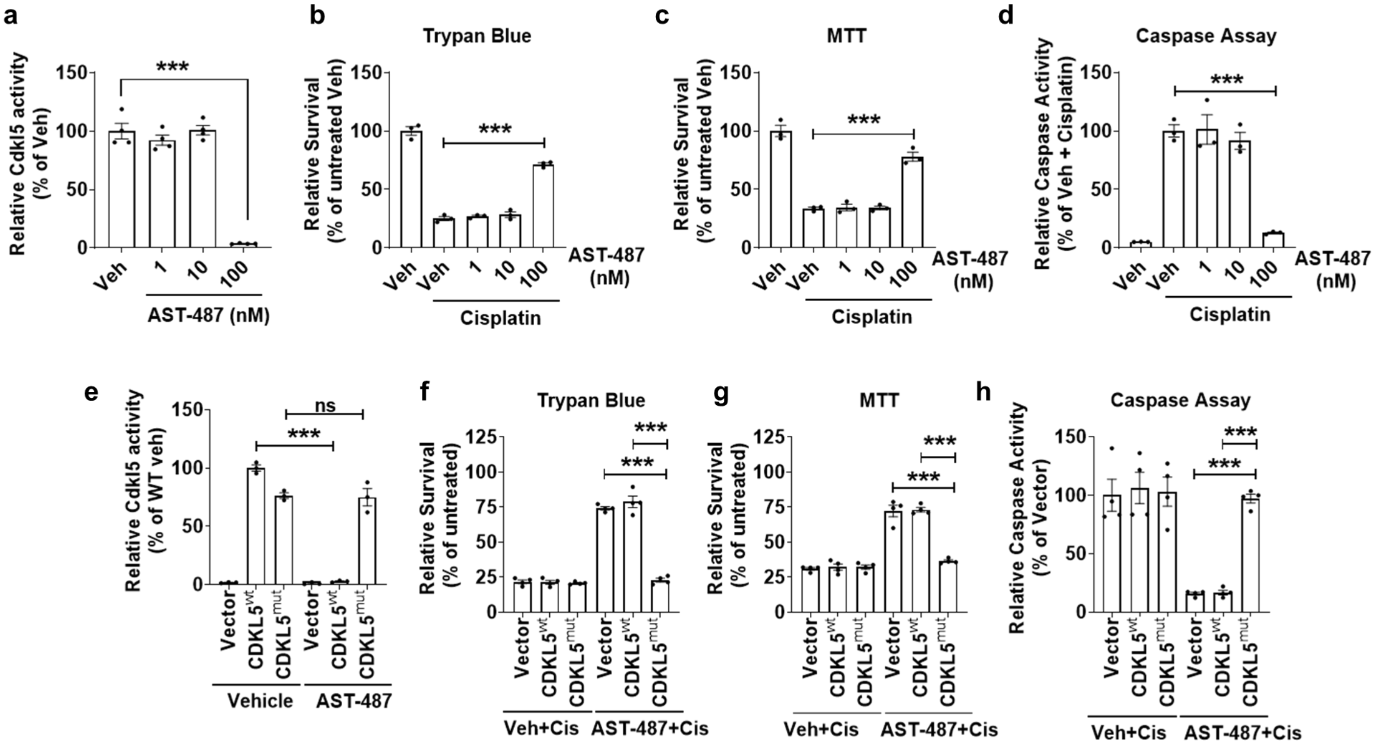

Supplementary Figure 18: AST-487 protects RTECs in a Cdkl5-dependent manner. (**a-d**) BUMPT cells were treated with 25 μM Cisplatin for 24 hours. At the same time, cells were co-treated with either vehicle or AST-487. After 24 hours, Cdkl5 kinase assay, cell viability and caspase activity assays were carried out, which showed that AST-487 protects BUMPT cells from cisplatin-associated cell death. (**e**) BUMPT cells were transfected with FLAG-tagged vector or CDKL5 WT or CDKL5 gatekeeper mutant (indicated as mut). After 24 hours, cell lysates were prepared followed by FLAG-immunoprecipitation and CDKL5 kinase assay in the presence or absence of 100 nM AST-487. The results indicate that the gatekeeper mutation confers resistance to AST-487 mediated CDKL5 inhibition. (**f-h**) BUMPT cells transfected with indicated constructs were treated with vehicle or 25 μM cisplatin and 100 nM AST-487 for 24 hours. After the incubation period, cell viability and caspase activity assays were carried out, which showed that AST-487 protects BUMPT cells from cisplatin-associated cell death in a CDKL5 dependent manner. Data are presented as individual data points (n = 3-5 biologically independent samples), from one out of 2-3 independent experiments, all producing similar results. In all the bar graphs, experimental values are presented as mean ± s.e.m. The height of error bar=1 s.e. and p<0.05 was indicated as statistically significant. 1-way ANOVA followed by Dunnett's (a-c) or Tukey's multiple-comparisons test (d-g) was carried out and statistical significance is indicated by *p < 0.05, **p < 0.01, ***p < 0.001. Source data are provided as a Source Data file.

Supplementary Figure 19

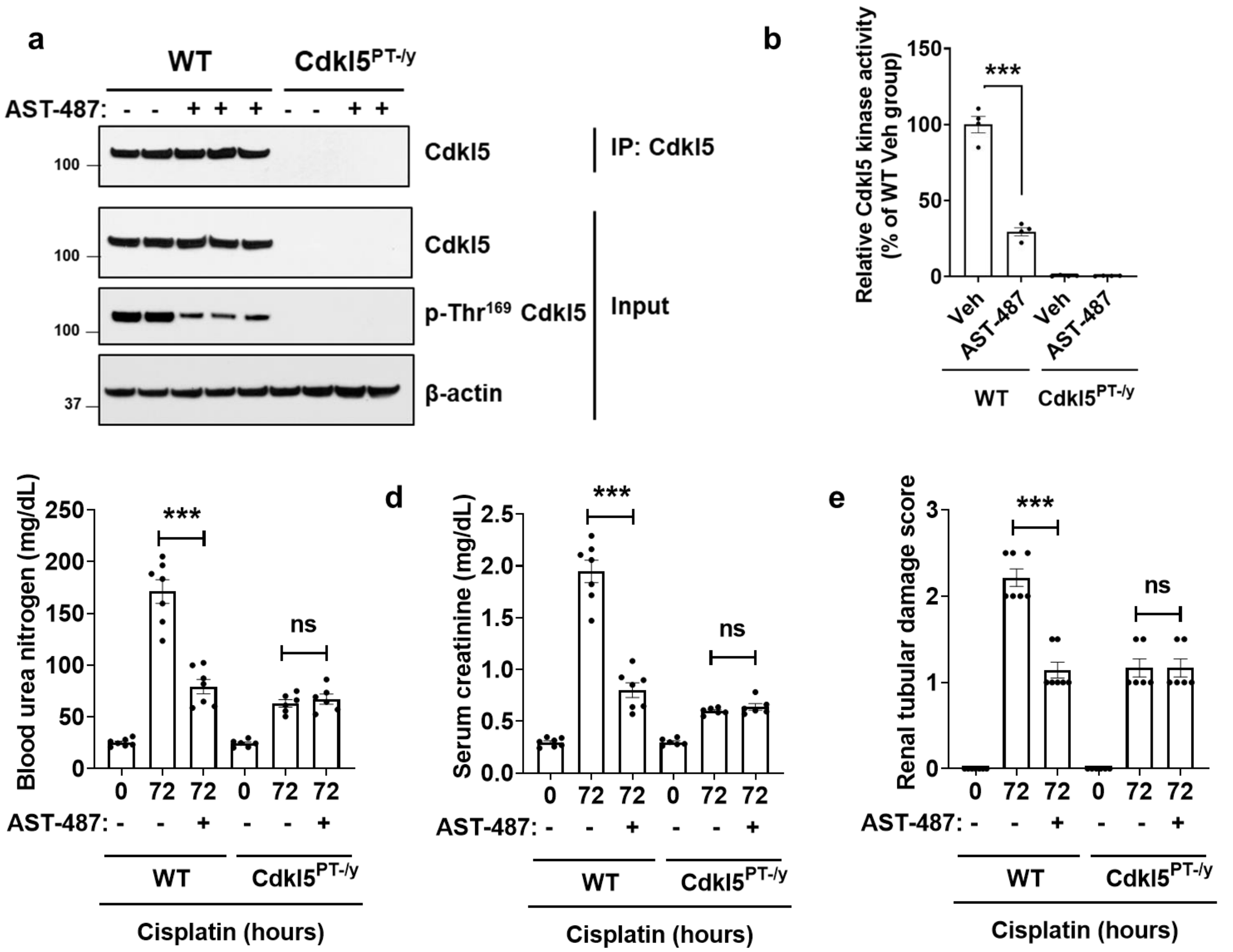

Supplementary Figure 19: AST-487 mediated renal protection is Cdkl5-dependent. Wild type and Cdkl5$^{PT-/y}$ mice were injected with cisplatin (30 mg/kg, i.p.) followed by treatment with either vehicle (-) or AST-487 (+). Subsequently, the renal tissues were collected after 24 hours followed by Cdkl5 immunoprecipitation, Cdkl5 kinase assays and immunoblot analysis of indicated proteins in the Cdkl5 immunoprecipitates and input lysates (whole tissue). (**a**) The representative immunoblots (input) show that AST-487 treatment resulted in reduced Cdkl5 phosphorylation in the TEY motif, while having no significant effect on total Cdkl5 protein levels. (**b**) Cdkl5 kinase assays showed that AST-487 can inhibit Cdkl5 activity in the renal tissues of the WT mice. Data presented (four biological replicates) are representative of two independent experiment that showed similar results. (**c-e**) Wild type and Cdkl5$^{PT-/y}$ mice were injected with cisplatin (30 mg/kg, i.p.) followed by treatment with either vehicle or AST-487 (25 mg/kg, oral gavage) 6 hours later, and subsequent assessment of renal function (BUN and serum creatinine) and histology (H&E, renal damage score) at indicated time-points. Data presented are cumulative of two independent experiments (n=7), that showed similar results. In all the bar graphs, experimental values are presented as mean ± s.e.m. The height of error bar=1 s.e. and $p<0.05$ was indicated as statistically significant. 1-way ANOVA followed by Dunnett's (b) or Tukey's multiple-comparisons test (c-e) was carried out and statistical significance is indicated by *$p < 0.05$, **$p < 0.01$, ***$p < 0.001$. Source data are provided as a Source Data file.

Supplementary Figure 20

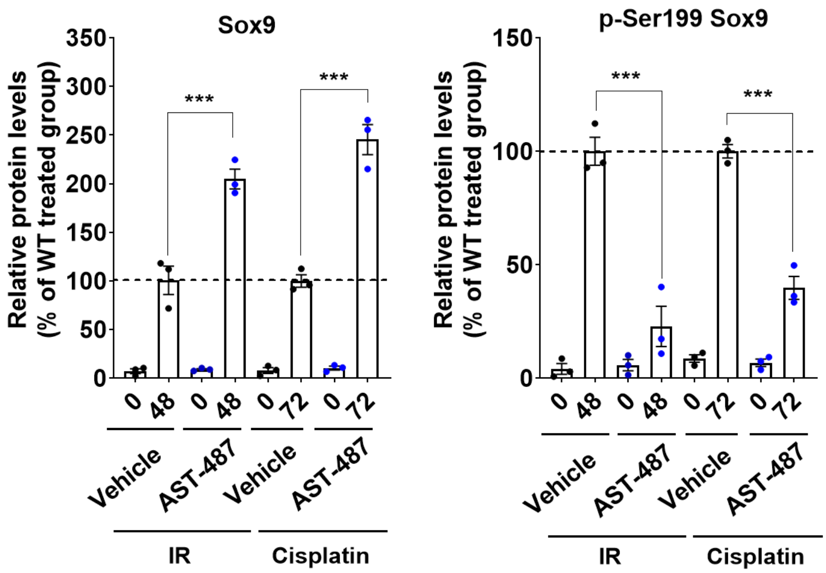

Supplementary Figure 20: AST-487 mediated Cdkl5 inhibition results in reduced Sox9 phosphorylation and increased Sox9 protein levels. Western blot analysis of renal tissues (representative blots shown in Figure 6i) indicated that AST-487 suppress Sox9 phosphorylation and increases Sox9 protein levels *in vivo*. Blots are representative of three independent experiments. In all the bar graphs, experimental values are presented as mean ± s.e.m. The height of error bar=1 s.e. and $p<0.05$ was indicated as statistically significant. 1-way ANOVA followed by Tukey's multiple-comparisons test was carried out and statistical significance is indicated by *$p < 0.05$, **$p < 0.01$, ***$p < 0.001$. Source data are provided as a Source Data file.

Supplementary Figure 21

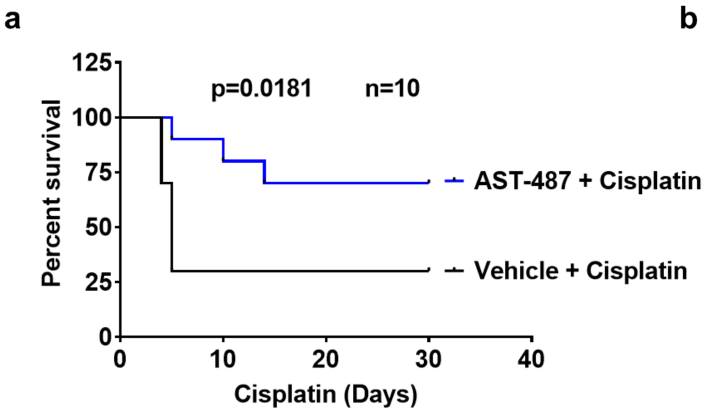

Supplementary Figure 21: Pharmacological or genetic Cdkl5 inhibition provides long-term protection from AKI. (**a**) Wild-type mice were treated with a single cisplatin (30 mg/kg) injection followed by administration with either vehicle or AST-487 (25 mg/kg, p.o.) and examination of overall survival up to 30 days. (**b**) Wild-type and *Cdkl5*$^{PT-/y}$ mice were treated with cisplatin (30 mg/kg) followed by examination of overall survival over a period of 30 days. Data is presented as Kaplan-Meier survival curves and Mantel-Cox test was performed to determine statistical significance. Source data are provided as a Source Data file.

Supplementary Figure 22

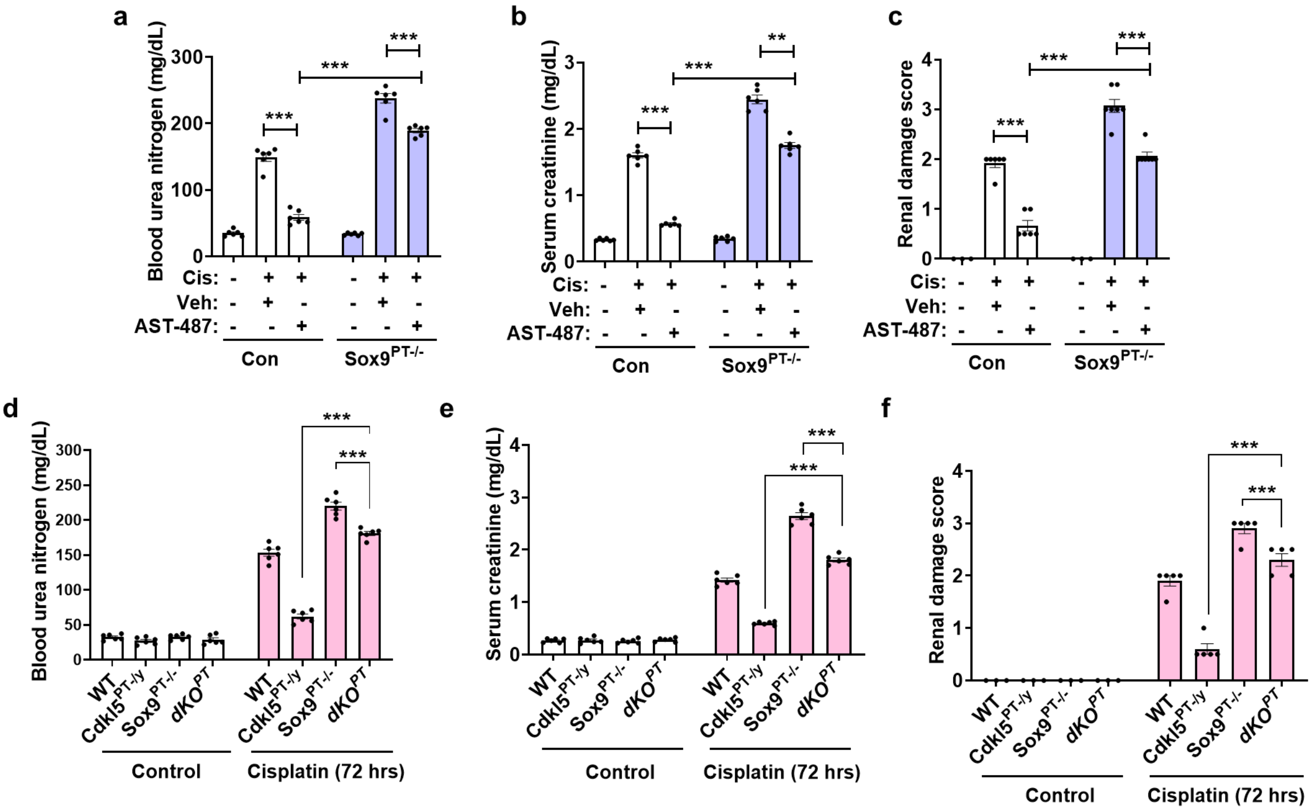

Supplementary Figure 22: Cdkl5 regulates cisplatin-associated AKI in a Sox9 dependent and independent manner. Littermate control and $Sox9^{PT-/-}$ were treated with cisplatin (30 mg/kg) followed by administration of either vehicle or AST-487 (25 mg/kg, oral gavage, 6 hours post-cisplatin injection). At 72 hours renal function and damage were assessed through (**a**) Blood urea nitrogen (**b**) Serum creatinine (**c**) renal histological analysis (H&E). Age-matched Wild type, $Cdkl5^{PT-/y}$, $Sox9^{PT-/-}$, and $Cdkl5^{PT-/y}Sox9^{PT-/-}$ (double knock out mice indicated as dKO^{PT}) were treated with cisplatin (30 mg/kg) followed by assessment of renal structure and function at 72 hours through (**d**) Blood urea nitrogen (**e**) Serum creatinine (**f**) renal histological analysis (H&E). Data presented (a-f) are cumulative of three independent experiment (n=6). In all the bar graphs, experimental values are presented as mean ± s.e.m. The height of error bar=1 s.e. and $p<0.05$ was indicated as statistically significant. 1-way ANOVA followed by Tukey's multiple-comparisons test was carried out and statistical significance is indicated by *p < 0.05, **p < 0.01, ***p < 0.001. Source data are provided as a Source Data file.

Supplementary Figure 23

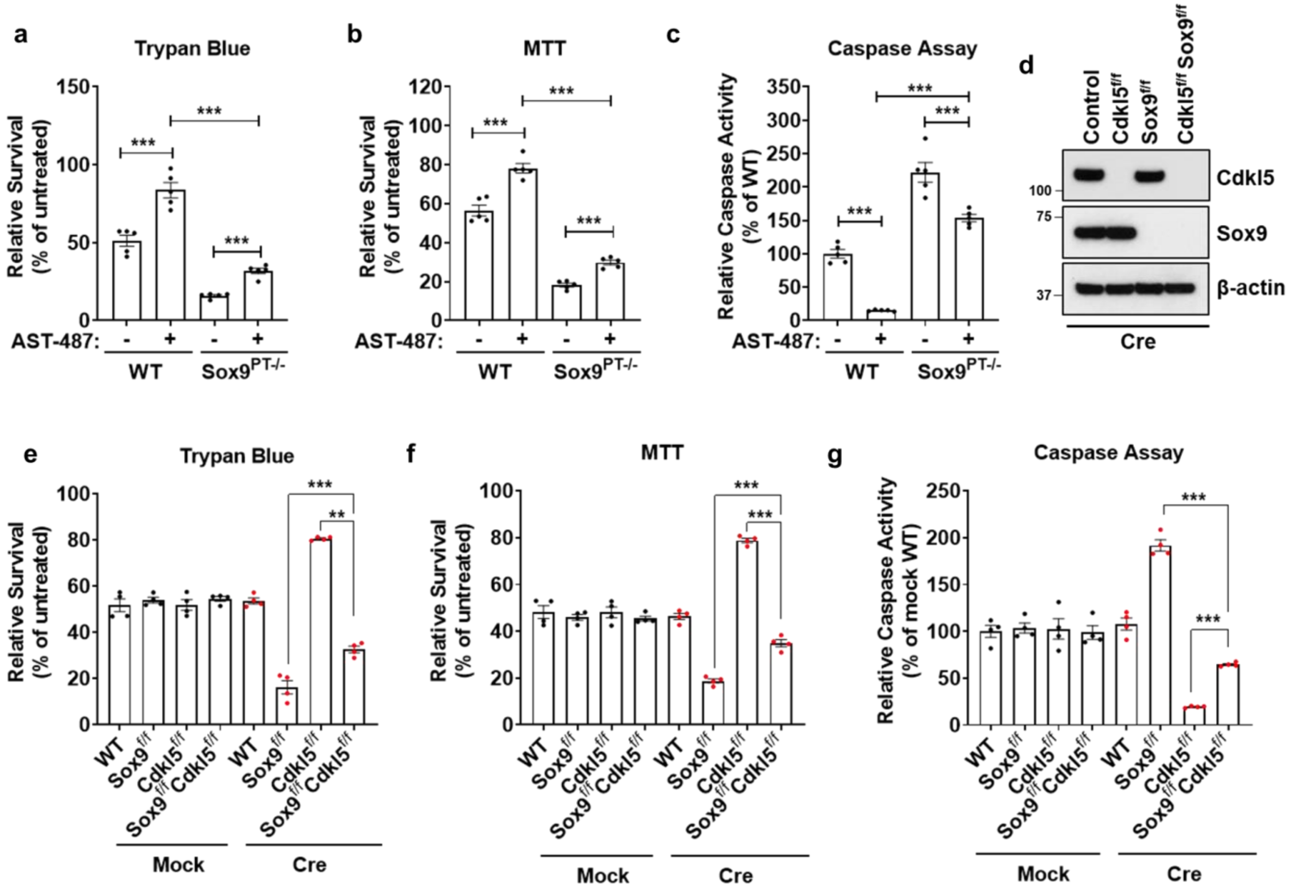

Supplementary Figure 23: Cdkl5 regulates cisplatin-associated RTEC cell death in a Sox9 dependent and independent manner. Primary murine RTECs were cultured from littermate control and Sox9$^{PT-/-}$ mice followed by co-treatment with 50 μM Cisplatin and either vehicle (-) or 100nM AST-487 (+) for 24 hours followed by assessment of cellular viability using Trypan Blue (**a**) and MTT (**b**) assays as well as Caspase activity (**c**). (**d**) Primary murine RTECs were cultured from Wild type control, Cdkl5$^{f/f}$, Sox9$^{f/f}$, and Cdkl5$^{f/f}$Sox9$^{f/f}$ mice. Subsequently RTECs were transduced with Cre expressing lentivirus and western blot analysis of untreated cells indicated successful knockout of floxed genes. (**e-g**) Primary murine RTECs from WT, Cdkl5$^{f/f}$, Sox9$^{f/f}$, and Cdkl5$^{f/f}$Sox9$^{f/f}$ mice were transduced with either control (mock) or Cre expressing lentivirus. Subsequently, the RTECs were treated with 50 μM Cisplatin for 24 hours followed by assessment of cell viability and caspase activity assay. Data presented are representative of three independent experiment (n=5-6 biological replicates). In all the bar graphs, experimental values are presented as mean ± s.e.m. The height of error bar=1 s.e. and $p<0.05$ was indicated as statistically significant. 1-way ANOVA followed by Tukey's multiple-comparisons test was carried out and statistical significance is indicated by *$p < 0.05$, **$p < 0.01$, ***$p < 0.001$. Source data are provided as a Source Data file.

Supplementary Figure 24

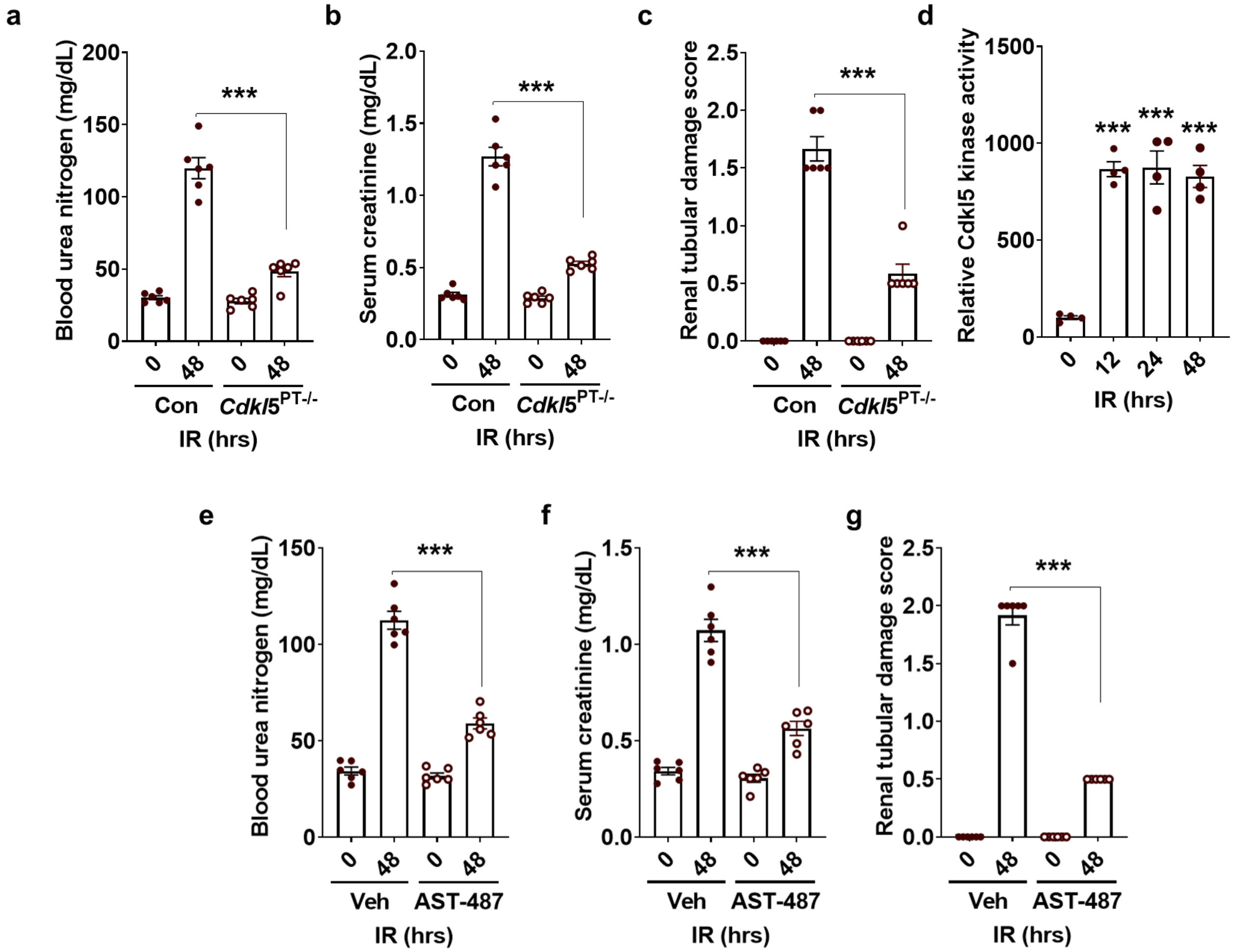

Supplementary Figure 24: Genetic or pharmacological Cdkl5 inhibition mitigates Ischemia-associated AKI in female mice. Control and Cdkl5$^{PT-/-}$ female littermates underwent bilateral renal ischemic surgery (35 minutes) followed by examination of renal damage. (**a**) Blood urea nitrogen (**b**) Serum creatinine (**c**) renal histological analysis (H&E) showed that Cdkl5 contributes to Ischemia-associated AKI. Data presented (a-c) are cumulative of three independent experiments (n=6) that showed similar results. (d) Renal tissue lysates were used for Cdkl5 immunoprecipitation followed by kinase assay at indicated time-points. The results showed that Cdkl5 activity increases during ischemic-AKI. The data presented is representative of two independent experiment (n=4 biological replicates). Eight weeks old wild type C57B6 female mice underwent bilateral renal ischemic surgery for 35 minutes, followed by either vehicle or 25 mg/kg (p.o.) AST-487 administration (6 hours post-IR) and subsequent examination of renal damage two days later. (**e**) Blood urea nitrogen (**f**) Serum creatinine (**g**) renal histological analysis (H&E) showed that Cdkl5 contributes to Ischemia-associated AKI. Data presented (e-g) are cumulative of three independent experiments (n=6) that showed similar results. . In all the bar graphs, experimental values are presented as mean ± s.e.m. The height of error bar=1 s.e. and p<0.05 was indicated as statistically significant. 1-way ANOVA followed by Dunnett's or Tukey's multiple-comparisons test was carried out and statistical significance is indicated by *p < 0.05, **p < 0.01, ***p < 0.001. Source data are provided as a Source Data file.

Supplementary Figure 25

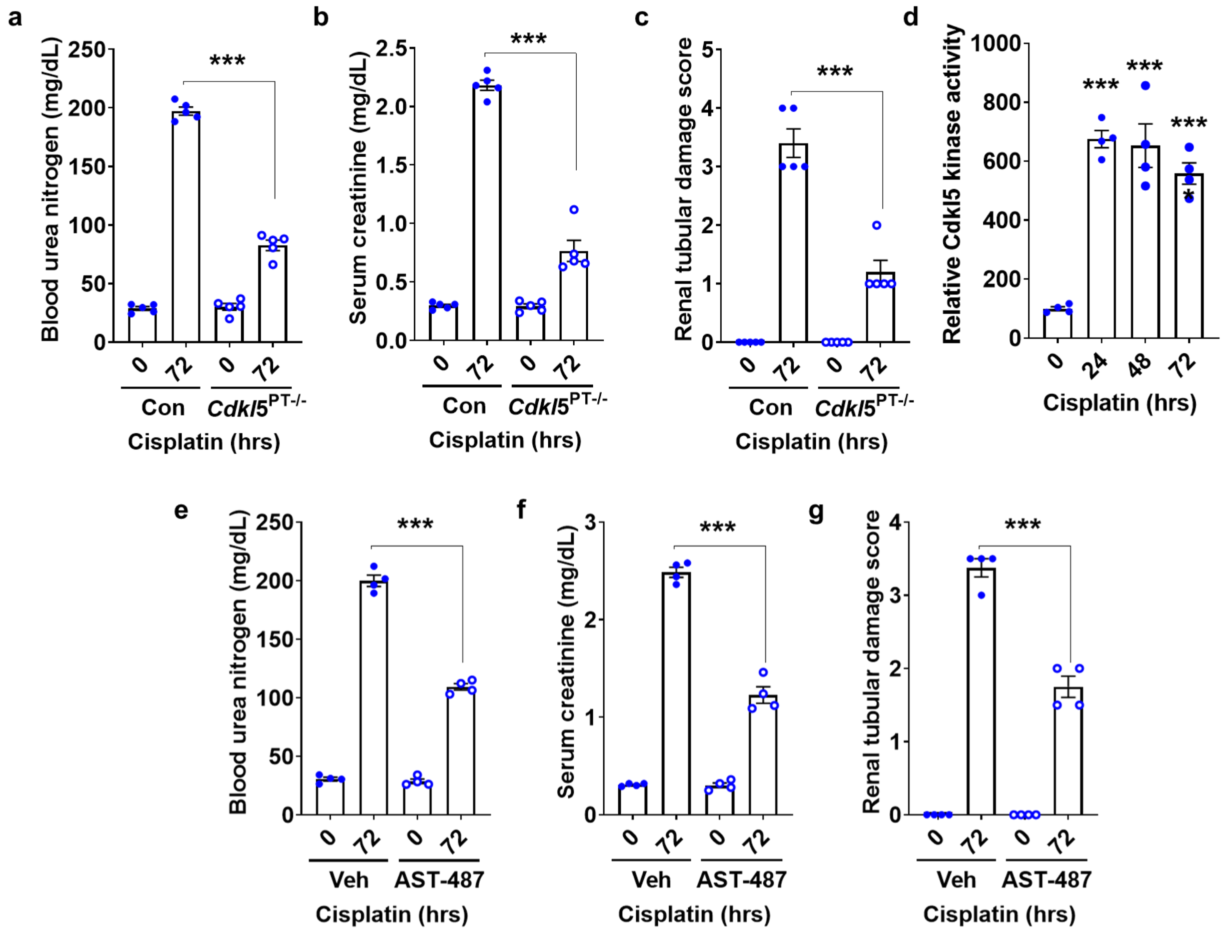

Supplementary Figure 25: Genetic or pharmacological Cdkl5 inhibition mitigates Cisplatin-mediated AKI in female mice. Control and Cdkl5$^{PT-/-}$ female littermates were treated with 15 mg/kg Cisplatin, followed by examination of renal damage. (**a**) Blood urea nitrogen (**b**) Serum creatinine (**c**) renal histological analysis (H&E) showed that Cdkl5 contributes to cisplatin-associated AKI. Data presented (a-c) are representative of two independent experiments (n=5) that showed similar results. (d) Renal tissue lysates were used for Cdkl5 immunoprecipitation followed by kinase assay at indicated time-points. The results showed that Cdkl5 activity increases during cisplatin nephrotoxicity. The data presented is representative of two independent experiment (n=4 biological replicates). Eight weeks old wild type C57B6 female mice were treated with 15 mg/kg Cisplatin, followed by either vehicle or 25 mg/kg (p.o.) AST-487 administration (6 hours post-cisplatin injection) and subsequent examination of renal damage three days later. (**e**) Blood urea nitrogen (**f**) Serum creatinine (**g**) renal histological analysis (H&E) showed that Cdkl5 contributes to Cisplatin-associated AKI. Data presented (e-g) are cumulative of representative of two independent experiments (n=4) that showed similar results. In all the bar graphs, experimental values are presented as mean ± s.e.m. The height of error bar=1 s.e. and $p<0.05$ was indicated as statistically significant. 1-way ANOVA followed by Dunnett's or Tukey's multiple-comparisons test was carried out and statistical significance is indicated by *$p < 0.05$, **$p < 0.01$, ***$p < 0.001$. Source data are provided as a Source Data file.

Supplementary Figure 26

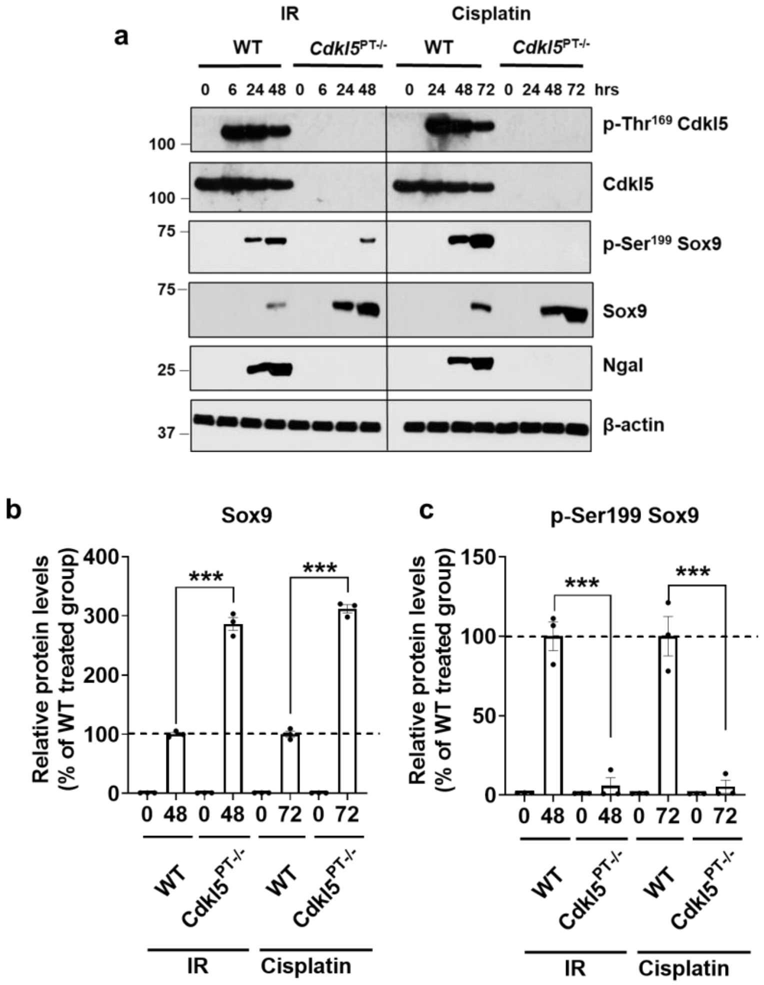

Supplementary Figure 26: Cdkl5 regulates Sox9 phosphorylation in female mice. Control, cisplatin and ischemic renal tissues from control and Cdkl5$^{PT-/-}$ female mice were subjected to immunoblot analysis of indicated proteins. Blots (**a**) are representative of three independent experiments. (**b**) Densitometric analysis of Sox9 and p-Ser-199 Sox9 protein levels (normalized to β-actin levels). Graph represents cumulative results (n=3 independent biological samples) from three independent experiments. In all the bar graphs, experimental values are presented as mean ± s.e.m. The height of error bar=1 s.e. and p<0.05 was indicated as statistically significant. 1-way ANOVA followed by Tukey's multiple-comparisons test was carried out and statistical significance is indicated by *p < 0.05, **p < 0.01, ***p < 0.001. Source data are provided as a Source Data file.

Supplementary Figure 27

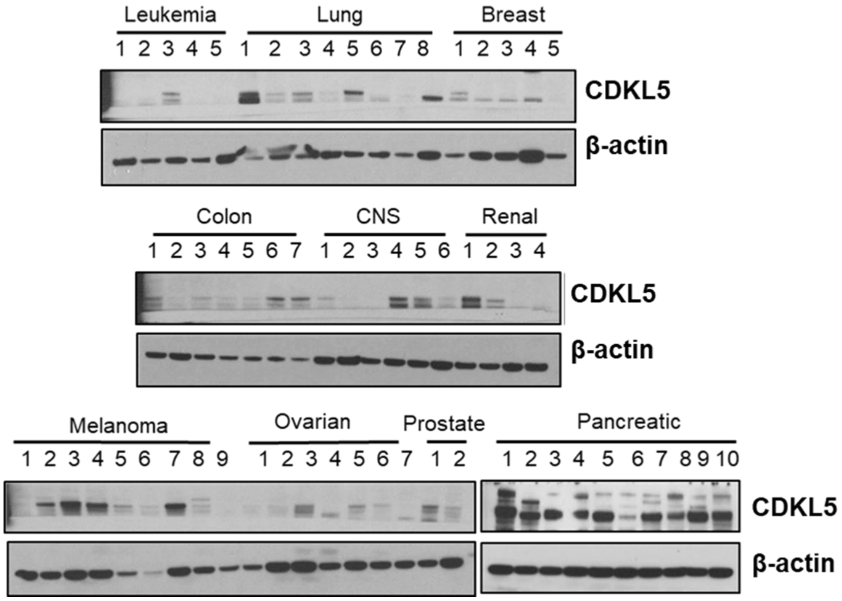

Supplementary Figure 27: CDKL5 is widely expressed in Cancer cell lines. Protein lysates from various cancer cell lines (NCI-60 and others) were subjected to immunoblot analysis of CDKL5 expression. CDKL5 was found to be widely expressed in most cell lines. The identity of cancer cell lines used here is provided in Supplementary File 2. Blots are representative of a single experiment. Source data are provided as a Source Data file.

Supplementary Table: 1

Hits obtained in the primary screen

BRAF	B-raf (rapidly accelerated fibrosarcoma) kinase
CDKL5	Cyclin-dependent like kinase 5
IGF1R	Insulin-like growth factor I receptor kinase
LIMK2	LIM domain kinase 2
MLKL	Mixed lineage kinase domain-like protein
MST2	Mammalian STE20-like kinase 2
TAO2	Thousand and one-amino acid protein kinase 2

Supplementary Table: 2

Sox9 mutagenesis primers

S64A-F	CCGGATCTGAAGAAGGAGGCCGAGGAAGATAAGTTCCC
S64A-R	GGGAACTTATCTTCCTCGGCCTCCTTCTTCAGATCCGG
S181A-F	CCGGCGGAGGAAGGCGGTGAAGAACGG
S181A-R	CCGTTCTTCACCGCCTTCCTCCGCCGG
S199A-F	CGGAACAGACTCACATCGCTCCTAATGCTATCTTC
S199A-R	GAAGATAGCATTAGGAGCGATGTGAGTCTGTTCCG
S211A-F	GCTGCAAGCCGACGCCCCACATTCCTC
S211A-R	GAGGAATGTGGGGCGTCGGCTTGCAGC
S412A-F	GCAGCAGCAGCACGCCCCGCAACAGAT
S412A-R	ATCTGTTGCGGGGCGTGCTGCTGCTGC
S199D-F	CCACGGAACAGACTCACATCGATCCTAATGCTATCTTCAAGG
S199D-R	CCTTGAAGATAGCATTAGGATCGATGTGAGTCTGTTCCGTGG

Cdkl5 mutagenesis primers

F89A-F	AGCATATTCTTCTCAACATATTCAGCCACCAGGTATAATTTTCCTCTCCG
F89A-R	CGGAGAGGAAAATTATACCTGGTGGCTGAATATGTTGAGAAGAATATGCT
Kinase Dead K42R-F	ACATGAAATTGTGGCAATCAGGAAATTCAAGGACAGCGAAG
Kinase Dead K42R-R	CTTCGCTGTCCTTGAATTTCCTGATTGCCACAATTTCATGT
TEY/AEY-F	CTCAGAGGGCAATAATGCTAATTATGCAGAGTTTGTGGCTACAAGG
TEY/AEY-R	CCTTGTAGCCACAAACTCTGCATAATTAGCATTATTGCCCTCTGAG
CA (1-340)-F	CTCACCACAGATCCCAGACGCGTACGCG
CA (1-340)-R	CGCGTACGCGTCTGGGATCTGTGGTGAG
CA (1-340)-R	CGCGTACGCGTCTGGGATCTGTGGTGAG
CA (1-340)-R	CGCGTACGCGTCTGGGATCTGTGGTGAG
CA (1-340)-R	CGCGTACGCGTCTGGGATCTGTGGTGAG

Supplementary Table: 2 (continued)

	Gene expression (qPCR) primers
Gadd45a	F: AGCAGAAGACCGAAAGGATGGACA
	R: AGCAGGCACAGTACCACGTTAT
Wwp2	F: CAGAGTCATTTGGATCTGAAG
	R: GTTGTGTGTTCTCCATTTTG
Sox9	F: CTCATTACCATTTTGAGGGG
	R: AAAATACTCTGGTTGCAAGG
Cdkl5	F: TTACTATTCAGAAGGTGCTAGG
	R: GGGTCCAACTTCAGTAAATTC
Kim1	F: TAATCACACTGAAGCAATCC
	R: TCAATCTTAGAGACACGGAAG
β-actin	F: GATGTATGAAGGCTTTGGTC
	R: TGTGCACTTTTATTGGTCTC
Myof	F: GGGAATTATAAACCCGGATAC
	R: ATCAAGAGAGGGAACAATCC
Sema3e	F: TATAGTGACCAGTGGTATTCAG
	R: TTGACGAACTAGAGGATGTG

Supplementary Table: 2 (continued)

	ChIP-qPCR primers
Ap2b-F	GGTGGGATTCAGCGTCCTTT
Ap2b-R	CTATTTCCACCGCGGCTCTA
Fzd9-F	GAAACAATGTGCCTGCCAGT
Fzd9-R	CAGCCTCTACTGTGGCCCTA
Gadd45a-F	CCGTGACTAGTAGGTTCAGGC
Gadd45a-R	CCTCGTAACTGCAAGCAGGA
Inhbb-F	AGGCCTGCTGAGGGCTCATTTCATC
Inhbb-R	CACACTTGCCTGCCACTCTCACTCG
Pi3kca-F	GCCTCTAGAAGTGCAAGCGT
Pi3kca-R	TGTCGGTAACTGTCGGAGGA
Ripk3-F	ACCTTGGTGGAGCAGAACTC
Ripk3-R	GCCAGGTACACATACTGGCT
S100a3-F	TGATGTGTGCGTTGCTAGGT
S100a3-R	AAGTAGGTCAACGCTGAGGC
S100A4-F	CAGGGCCTGGTGCTTGTGGTT
S100A4-R	GTGCCTCTCAGTCCTGGCCTCAACA
Sulf2-F	TCACGCCTAATTGCGGGCTGGTGT
Sulf2-R	CCCATGGGCACGGGAAGTGTGG
Timp2-F	CTGAGTTCCACCCAGAGTCG
Timp2-R	CACTCACACACGAAAACGGC
Wwp2-F	CTGGCAAATGAGTCTCCTCCACAAC
Wwp2-R	GGCCGTGATTGACTACTTTGATGTC
Adamts14-F	TTCCTTGCACTACCGTGCTC
Adamts14-R	ACAGCCCAACTGCTCGTATG
Dusp6-F	ACACAGGTGGTTTTCCGTCC
Dusp6-R	GTTGGGAAGTCTCATGGGCA
Epha2-F	CCTGGTGGCGCTTTGAAATG
Epha2-R	TTTAAAGGGGCCGGACTGAC
Fkbp5-F	CAGCCATCCTTTGGGTTCCT
Fkbp5-R	TGCGATGTAGCACCTAGCAC
Myof-F	CGAGACTCGGTAAAACAGTGC
Myof-R	AAGTTTTGCGCAGCCAATGA
Nedd9-F	TACAAATCATGTCCCGCGCT
Nedd9-R	TCTCTCCCCCAAATGACCCA
Nek8-F	AAACAACCCTTCTCTCGCCC
Nek8-R	CTGCTAGGGTTTCGACCAGG
Sema3e-F	ACAGACCCTGCCCTTAGGTA
Sema3e-R	TAAAAGCCAGGGAGATGGGC
Sparc-F	AGACCTAGAGGGGACTTCGC
Sparc-R	GCACTCTCTTGGGCTTAGCA

Supplementary Table: 3

Cell Line	Tuomr Classfication	Blot ID
HL-60	Leukemia	1
K-562	Leukemia	2
MOLT-4	Leukemia	3
RPMI-8226	Leukemia	4
SR	Leukemia	5
EKVX	Non-small Cell Lung	1
HOP-62	Non-small Cell Lung	2
HOP-92	Non-small Cell Lung	3
NCI-H226	Non-small Cell Lung	4
NCI-H23	Non-small Cell Lung	5
NCI-H322M	Non-small Cell Lung	6
NCI-H460	Non-small Cell Lung	7
NCI-H522	Non-small Cell Lung	8
MCF-7	Breast	1
MDA-MB-231	Breast	2
MDA-MB-468	Breast	3
HS 578T	Breast	4
T-47D	Breast	5
COLO205	Colon	1
HCC-2998	Colon	2
HCT-116	Colon	3
HCT-15	Colon	4
HT29	Colon	5
KM12	Colon	6
SW-620	Colon	7
SF-268	CNS	1
SF-295	CNS	2
SF-539	CNS	3
SNB-19	CNS	4
SNB-75	CNS	5
U251	CNS	6
RXF 393	Renal	1
SN12C	Renal	2
TK-10	Renal	3
UO-31	Renal	4
LOX IMVI	Melanoma	1
MALME-3M	Melanoma	2
M14	Melanoma	3
MDA-MB-435	Melanoma	4
SK-MEL-2	Melanoma	5
SK-MEL-28	Melanoma	6
SK-MEL-5	Melanoma	7
UACC-257	Melanoma	8
UACC-62	Melanoma	9
IGR-OV1	Ovarian	1
OVCAR-3	Ovarian	2
OVCAR-4	Ovarian	3
OVCAR-5	Ovarian	4
OVCAR-8	Ovarian	5
NCI/ADR-RES	Ovarian	6
SK-OV-3	Ovarian	7
PC-3	Prostate	1
DU-145	Prostate	2
HPDE	Pancreatic	1
HPAF II	Pancreatic	2
L3.6 PL	Pancreatic	3
CAPAN-1	Pancreatic	4
PANC-1	Pancreatic	5
MIAPACA-2	Pancreatic	6
CFPAC-1	Pancreatic	7
SW1990	Pancreatic	8
PANC 10.05	Pancreatic	9
SU86.86	Pancreatic	10

Supplementary Table: 4

Position	Peptide (with modification)	Unique	z	obs m/z	SP	PPM	xCorr	Delta Corr
83-94	K.GYDWTLVPMPVR.V + Oxidation (M)	Y	2	716.862	834.56	0.84	4.667	0.481
107-120	K.RPMNAFMVWAQAAR.R + Oxidation (M)	Y	2	824.41	1309.4	0.92	5.136	0.563
123-137	K.LADQYPHLHNAELSK.T	Y	2	867.938	968.2	1.67	5.309	0.471
184-205	K.NGQAEAEEATEQTHISPNAI FK.A	Y	3	795.044	1611.3	0.71	4.895	0.431
397-435	K.TEQLSPSHYSEQQQHSPQQI SYSPFNLPHYSPSYPPITR.S	Y	3	1518.72	1963.45	1.21	3.954	0.628

Supplementary Table 4. Unique polypeptides of Sox9 identified by LC-MS/MS analysis of in-gel trypsin digestion of protein band coimmuniprecipitated with CDKL5.
